# The mitochondrial genome of *Heterosentis pseudobagri* (Wang & Zhang, 1987) Pichelin & Cribb, 1999 reveals novel aspects of tRNA genes evolution in Acanthocephala

**DOI:** 10.1186/s12864-023-09177-9

**Published:** 2023-03-02

**Authors:** Jin-Wei Gao, Xi-Ping Yuan, Ivan Jakovlić, Hao Wu, Chuan-Yu Xiang, Min Xie, Rui Song, Zhong-Gui Xie, Yuan-An Wu, Dong-Sheng Ou

**Affiliations:** 1 Hunan Fisheries Science Institute, 728 Shuanghe Rd, Kaifu District, Changsha, 410153 Hunan China; 2 State Key Laboratory of Grassland Agro-Ecosystems, and College of Ecology, Lanzhou University, Lanzhou, 730000 China

**Keywords:** Acanthocephala, Arhythmacanthidae, Mitogenome, mtDNA, Transcriptome, Posttranscriptional tRNA editing, Truncated tRNA, Transport RNA

## Abstract

**Background:**

Acanthocephala is a clade of obligate endoparasites whose mitochondrial genomes (mitogenomes) and evolution remain relatively poorly understood. Previous studies reported that *atp8* is lacking from acanthocephalan mitogenomes, and that tRNA genes often have nonstandard structures. *Heterosentis pseudobagri* (Arhythmacanthidae) is an acanthocephalan fish endoparasite for which no molecular data are currently available, and biological information is unavailable in the English language. Furthermore, there are currently no mitogenomes available for Arhythmacanthidae.

**Methods:**

We sequenced its mitogenome and transcriptome, and conducted comparative mitogenomic analyses with almost all available acanthocephalan mitogenomes.

**Results:**

The mitogenome had all genes encoded on the same strand and unique gene order in the dataset. Among the 12 protein-coding genes, several genes were highly divergent and annotated with difficulty. Moreover, several tRNA genes could not be identified automatically, so we had to identify them manually via a detailed comparison with orthologues. As common in acanthocephalans, some tRNAs lacked either the TWC arm or the DHU arm, but in several cases, we annotated tRNA genes only on the basis of the conserved narrow central segment comprising the anticodon, while the flanking 5’ and 3’ ends did not exhibit any resemblance to orthologues and they could not be folded into a tRNA secondary structure. We corroborated that these are not sequencing artefacts by assembling the mitogenome from transcriptomic data. Although this phenomenon was not observed in previous studies, our comparative analyses revealed the existence of highly divergent tRNAs in multiple acanthocephalan lineages.

**Conclusions:**

These findings indicate either that multiple tRNA genes are non-functional or that (some) tRNA genes in (some) acanthocephalans might undergo extensive posttranscriptional tRNA processing which restores them to more conventional structures. It is necessary to sequence mitogenomes from yet unrepresented lineages and further explore the unusual patterns of tRNA evolution in Acanthocephala.

**Supplementary Information:**

The online version contains supplementary material available at 10.1186/s12864-023-09177-9.

## Background

Acanthocephala is a clade of obligate endoparasites. Traditionally a stand-alone phylum, in more recent classifications it forms phylum Syndermata together with Rotifera (Protostomia: Spiralia: Gnathifera: Syndermata) [1, 2]. All known acanthocephalans, or thorny-headed worms, are endoparasites with a complex lifecycle including at least two hosts: commonly an arthropod (Mandibulata) intermediate host and a vertebrate (Gnathostomata) definitive host [3]. The definitive host range is very wide, including amphibians, birds, and mammals, but the most common hosts are bony fishes (Osteichthyes). Commonly, the adults parasitize in the intestinal tract of the host, where the attachment of the parasite to the intestinal wall via a hooked or spined proboscis can damage the host’s intestinal epithelium. The consequences of these injuries are more often lethal in mammals than in fish [1].

Acanthocephalan species belonging to the genus *Heterosentis* (Palaeacanthocephala: Echinorhynchida: Arhythmacanthidae) parasitize both freshwater and marine fish and they have a very wide geographic distribution [4–6]. *Heterosentis* (syn. *Arhythmacanthus*) *pseudobagri* (Wang & Zhang, 1987) Pichelin & Cribb, 1999 is a poorly researched fish endoparasite that commonly parasitizes in the intestines of *Tachysurus* (syn. *Pelteobagrus*) *fulvidraco* (Richardson, 1846) (Bagridae) [7]. The few studies that have researched this species are rather old and published in Chinese language [7, 8]. As a result of this neglect, currently there are no molecular data for this species, and data about its biology are not available to the international scientific audience. Moreover, there are only a handful of sequences available for the entire Arhythmacanthidae family, and molecular data are available only for only two species from this genus: *Heterosentis holospinus* Amin, Heckman & Ha, 2011 [5] and an unidentified *Heterosentis* sp. [9]. Morphological identification of fish parasites is often fraught with difficulties due to the low number of suitable morphologic traits and common host-induced morphological variability [10–12]. Indeed, strong host-induced intraspecific morphological variability was also observed in some acanthocephalans [13]. It is, therefore, necessary to integrate morphological and molecular data both for identification and phylogenetic studies of parasites [14]. In addition, due to the scarcity of molecular data, many aspects of the taxonomy and phylogeny of Acanthocephala, including the phylogenetic position of the family Arhythmacanthidae, remain debated [1, 2, 9, 15–19].

Mitochondrial genomes (mitogenomes) of bilaterian animals are usually very compact and highly conserved in terms of gene content and organisation: a circular molecule encoding 37 genes (13 protein-coding genes, 2 rRNA genes and 22 tRNA genes) [20, 21]. However, some bilaterian invertebrate lineages exhibit certain unusual architectural features, including fragmented or linearized mitogenomes, or loss of some of the genes [21–24]. Several major lineages appear to have lost *atp8* from their standard repertoire, and some have lost some of the tRNA genes [23–28]. Previous studies indicated that *atp8* is also probably lacking from acanthocephalan mitogenomes, but the tRNA gene repertoire appears to be standard (22 genes) [24, 29, 30]. However, multiple previous studies reported that acanthocephalan tRNA genes often do not exhibit the standard cloverleaf structure as they commonly lack either the TWC arm or the DHU arm, or even both [15, 29–32]. Previous studies also reported high gene order (tRNA) rearrangement rates [15].

Mitogenomes are currently (April 2022) available for only 22 acanthocephalan species, and none for the family Arhythmacanthidae. As this is only ≈2% of acanthocephalan species known to science [1], mitogenomic evolution in Acanthocephala remains rather poorly understood. To address this dearth of molecular data for *H. pseudobagri* and Acanthocephala, we sequenced the complete mitochondrial genome and two partial nuclear genes (*18S* and *28S*) of *H. pseudobagri* and used the data to conduct comparative mitogenomic analyses of all available acanthocephalan mitogenomes. This study shall greatly facilitate future identification of this species, taxonomic and phylogenetic studies of Acanthocephala, as well as contribute to our understanding of mitogenomic evolution in Acanthocephala.

## Results

### Morphology, identity and phylogeny

**Fig. 1:**
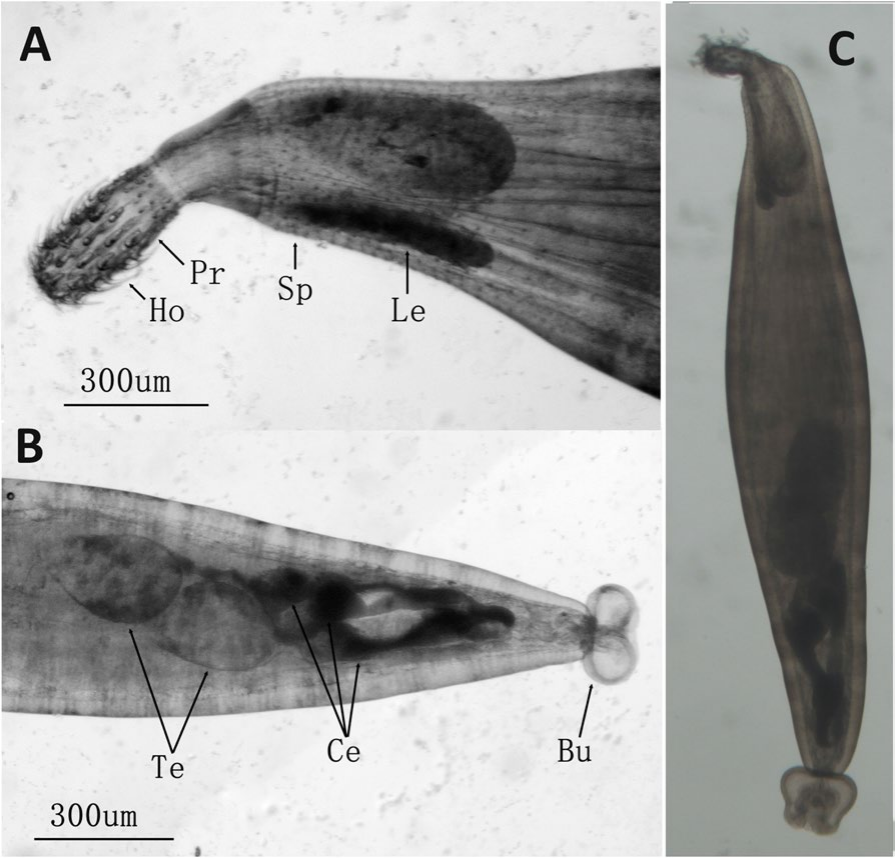
Morphology of *Heterosentis pseudobagri*. **A** A microscopic image of the anterior region of the sampled parasite. Visible are proboscis hooks (Ho), proboscis (Pr), spines (Sp), Lemnisci (Le). The size bar is shown below. **B** A microscopic image of the central and posterior region of the sampled parasite. Visible are the male reproductive system – testes (Te), Cement gland (Ce) and Bursa (Bu). The size bar is shown below. **C** A microscopic image of the entire parasite specimen

The species was identified morphologically according to the original description [8]: trunk cylindrical; anteriorly 50 circles of spines, 20 spines in each circle; the proboscis is small, more or less cylindrical, longer than wide, with 5 circular rows of 12 rooted hooks each; the proboscis hooks have roots, the anterior 3 circles of hooks are robust, almost the same in size, whereas the posterior two circles are small; the cement glands (6 in total) are clavate; the copulatory bursa is reversible (Fig. 1). The sampled specimen was male, with two oval, partially overlapping testes. The original description stated that the lemniscus (2 in total) is longer than the receptacle of the proboscis, but in our specimen both lemniscs are about the same length as the receptacle, or even slightly shorter. Due to the scarcity of data for this species, it is unclear whether this inconsistency was caused by the fixation method, or whether this trait is highly variable in this species.

**Fig. 2:**
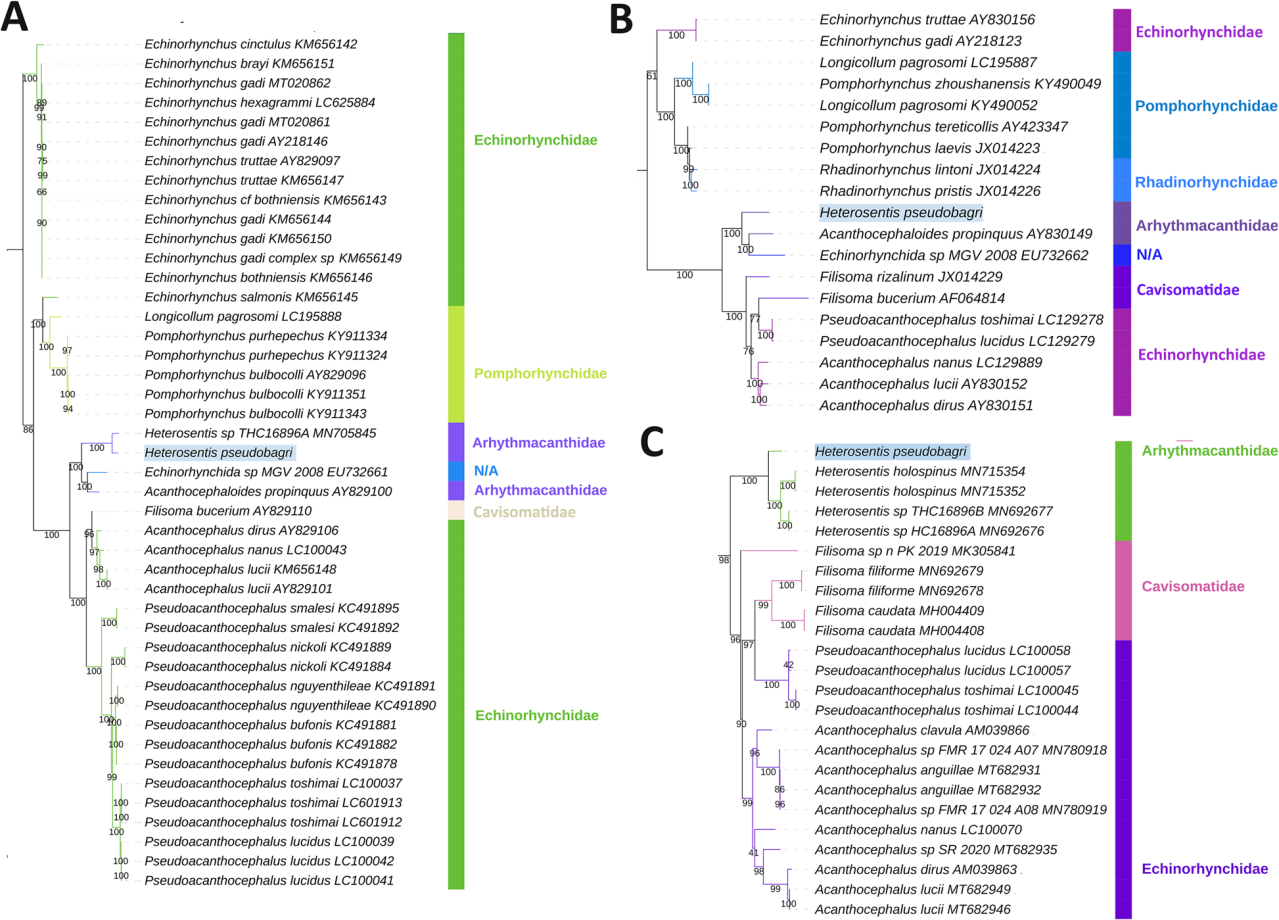
Single-gene phylogenetic analyses: **A**) *28S*, **B**) *18S*, **C**) *cox1*. Numbers at nodes indicate bootstrap support, *H. pseudobagri* is highlighted using colour shading, and family-level identity is shown to the right (also indicated by branch colour)

As there are no molecular data available for this species, we could not identify it at the species level using molecular tools. However, in all three single-gene analyses (*cox1*, *18S* and *28S*), the studied species formed a clade with other Arhythmacanthidae with high support, and in *cox1* and *28S* analyses, it formed a clade with other *Heterosentis* species (Fig. 2). This being the first sequenced arhythmacanthid mitogenome, mitochondrial phylogenomics could only identify it as a member of (paraphyletic) Echinorhynchida (Additional file 1: Figures S1-S3). All three single-gene analyses conducted herein resolved Arhythmacanthidae as closely related to the sister-clade of Cavisomatidae + Echinorhynchidae (Fig. 2). These results are in full congruence with the few previous studies that included data from this family, but these studies also relied on the same three genes, i.e. *18S* and *28S* [9] and *cox1* [5]. Furthermore, most families were deeply paraphyletic in single-gene analyses. We conducted three mitogenomic analyses: maximum likelihood analysis (ML) of nucleotide sequences of all 12 PCGs; Bayesian inference analysis of the same dataset; and an ML analysis of the same dataset but with 3^rd^ codon sites removed. All three analyses produced identical topologies, where Arhythmacanthidae + Pomphorhynchidae were sister families, which then formed a clade together with Echinorhynchidae (Additional file 1: Figures S1-S3). The latter node had a strong posterior probability (1.0) support in the BI analysis, weak SH-aLRT support (78.7%) in the ML analysis, and very low support (50.6%) when the third codon position was removed from the alignment. Cavisomatidae remains unavailable in the mitogenomic dataset. These three families formed the main clade of the order Echinorhynchida, which was rendered paraphyletic due to the placement of Illiosentidae + Rhadinorhynchidae at the base (sister group to all other taxa) of the Palaeacanthocephala clade.

### Comparative mitogenomic architecture

The mitogenome of *H. pseudobagri* was PCR-amplified and Sanger-sequenced from extracted tissue DNA. It had a circular structure with all genes encoded on the same strand (Additional file 1: Figure S4). At 13,742 bp, it was among the smallest available acanthocephalan mitogenomes, which ranged in size from 13,393 to 15,884 bp, and exhibited a relatively small standard deviation in size (14,816 ± 661 bp) (Additional file 2: panel A). This was also reflected in only one large (> 100 bp) noncoding region (NCR): 480 bp between *trnW* and *trnE*. Most other acanthocephalan species possessed two or more large NCRs (Additional file 1: Figure S5), but the second largest NCR in the mitogenome of *H. pseudobagri* was just below the selected threshold of 100 bp: 98 bp between *trnQ* and *trnY*. Other NCRs were all below 40 bp in size, and there were only nine intergenic regions in total (Table 1). Gene sizes in the mitogenome of *H. pseudobagri* did not exhibit any outlier values, but gene sizes had relatively wide ranges in the acanthocephalan dataset (Additional file 2: panel B). rRNA genes were 909 bp (*rrnL*) and 722 bp (*rrnS*) in size. The mitogenome exhibited a remarkably large number of putative gene overlaps (19). These were also unusually large (ranging from 1 to 28 bp), with ten overlaps ≥ 10 bp. Although these overlaps may be footprints of selection for small size, we suspect that many of them are likely to be annotation artefacts caused by truncated tRNA genes (discussed in more detail in the ‘tRNAs’ section). As regards the base composition, its A + T content of 62.5% was standard for this dataset (54.5 to 68.3%), but it had a very high T content of 40.9%. This was the second-highest value in the entire dataset, with only *Paratenuisentis ambiguus* exhibiting a higher value (42.1%). In other aspects, its base composition was not unique within the dataset. The GC skew (on the entire coding strand) was 0.561, which is also average for Acanthocephala (0.39 to 0.68 in the currently available dataset; Additional file 2: panel A). Start codons were standard: the most common was GTG (8 genes), followed by TTG (3 genes), and ATA (only in *nad1*). Stop codons were also standard: TAG (5 genes), TAA (4 genes), and the abbreviated T–, common in acanthocephalans, was also found in four genes (Table 1, Additional file 2: panel B).

**Table 1:** The architecture of the mitogenome of *H. pseudobagri*. IGN/O shows intergenic regions (positive values) and overlaps (negative values)

Gene	Position		Size	IGN/O	Codon		Anticodon
	From	To			Start	Stop	
cox1	1	1557	1557		GTG	TAG	
trnG	1540	1591	52	-18			TCC
trnQ	1580	1643	64	-12			TTG
trnY	1742	1793	52	98			GTA
rrnL	1794	2702	909				
trnL1	2703	2772	70				TAG
nad6	2755	3175	421	-18	GTG	T–	
trnI	3175	3225	51	-1			GAT
trnD	3217	3286	70	-9			GTC
atp6	3322	3882	561	35	GTG	TAG	
nad3	3873	4241	369	-10	TTG	TAA	
trnW	4222	4283	62	-20			TCA
NCR	4284	4763	480				
trnE	4764	4815	52				TTC
trnV	4822	4882	61	6			TAC
trnT	4886	4946	61	3			TGT
trnS2	4937	4985	49	-10			TGA
nad4L	4983	5240	258	-3	GTG	TAG	
nad4	5213	6505	1293	-28	GTG	TAG	
trnH	6502	6551	50	-4			GTG
nad5	6543	8193	1651	-9	GTG	T–	
trnL2	8194	8246	53				TAA
trnP	8270	8320	51	23			TGG
cytb	8321	9448	1128		GTG	TAG	
nad1	9481	10,342	862	32	ATA	T–	
trnR	10,342	10,404	63	-1			TCG
trnK	10,394	10,447	54	-11			CTT
trnM	10,456	10,510	55	8			CAT
rrnS	10,511	11,232	722				
trnF	11,225	11,275	51	-8			GAA
cox2	11,276	11,911	636		GTG	TAA	
trnC	11,902	11,942	41	-10			GCA
cox3	11,936	12,653	718	-7	TTG	T–	
trnA	12,654	12,706	53				TGC
trnN	12,736	12,792	57	29			GTT
trnS1	12,790	12,845	56	-3			ACT
nad2	12,828	13,739	912	-18	TTG	TAA	

The newly sequenced *H. pseudobagri* exhibited a unique gene order, but it diverged from other acanthocephalan mitogenomes only in terms of tRNA gene arrangement (Additional file 1: Figure S6). Major differences in comparison to other species were observed in the large tRNA box comprising W-NCR-(M)-V-K-E-T-S2 genes in many other species: in *H. pseudobagri* the arrangement of this box was W-NCR-E-V-T-S2.

We managed to annotate the complete set of standard acanthocephalan genes in the mitogenome, 2 rRNA genes, 12 protein-coding genes (PCGs), and 22 tRNA genes, but the annotation of several genes was not straightforward. Several PCGs were highly divergent (*nad6, nad4L*…), which made their annotation very difficult, and several tRNA genes could not be identified automatically using MITOS and ARWEN tools (*trnH, trnQ, trnM, trnD, trnN, trnI,* and *trnR)*, so we had to identify them manually via a detailed comparison with orthologues. To corroborate that this was not caused by sequencing artefacts, we sequenced the transcriptome of another conspecific specimen and assembled the mitogenome from transcriptomic data.

### Mitogenome assembly from transcriptomic data

The transcriptomic mitogenome assembly consisted of 263 contigs, with a total size of 124,638 bp. The N50 of the assembly was 591 bp. The largest contig was 9,096 bp, but only 19 contigs were larger than 1,000 bp. Twenty-five contigs contained multiple genes (Table 2). Among these, most contigs contained two to three genes, five contigs contained four genes, and contig 239 was a major outlier with 11 genes: *trnE, nad4, nad5, trnL, trnP, cytb, nad1, trnF, cox2, cox3,* and *trnS*. There were several contigs where genes were mistakenly identified on the minus strand, which is indicative of contig assembly artefacts. In most cases the order of genes corresponded to the order on the mitogenome, but many tRNA genes were omitted (i.e. not annotated). Surprisingly, even some PCGs were not identified in a few cases: *nad4L*, *nad3*, and *nad6* (e.g. contigs 4, 241, 242, etc.). The assembled transcriptomic assembly was mostly identical to the DNA assembly, except for several different bases at the 5’ end of *cox1*, a few SNPs, and an unsequenced stretch between positions 4,610 and 4,669, corresponding to the large NCR.

**Table 2:** Contigs of the transcriptomic mitogenome assembly that contained multiple genes. The strand on which genes are encoded is shown in brackets after the contig number. In a few cases where genes within a single contig were annotated on different strands, the strand is shown next to the gene name. Contig 239 was an outlier with 11 encoded genes, so they are listed in the text

contig No. (strand)	genes			
contig 1 ( +)	trnF	cox2	cox3	
contig 12 ( +)	cox2	cox3		
contig 13	cox3 (-)	cox3 ( +)		
contig 2	trnG (-)	cox1 (-)	trnG ( +)	
contigs 246, 247, 249 ( +)	cox1	trnG		
contig 248 (-)	trnG	cox1		
contig 3 (-)	trnT	trnV	trnE	
contigs 4 and 5 ( +)	trnE	trnV	trnT	nad4
contig 29 ( +)	trnE	trnV	trnT	
contigs 6 and 8 (-)	cytb	trnP	trnL	
contig 7 ( +)	nad4	nad5		
contig 10 (-)	trnS	trnA	cox3	
contig 11 (-)	trnL	rrnL		
contig 40 ( +)	rrnL	trnL		
contig 240 (-)	trnW	atp6	rrnL	
contig 241, 244 and 245 (-)	trnW	atp6	trnL	rrnL
contigs 242 and 243 ( +)	rrnL	trnL	atp6	trnW
contig 239 ( +)	11 genes			

**Fig. 3:**
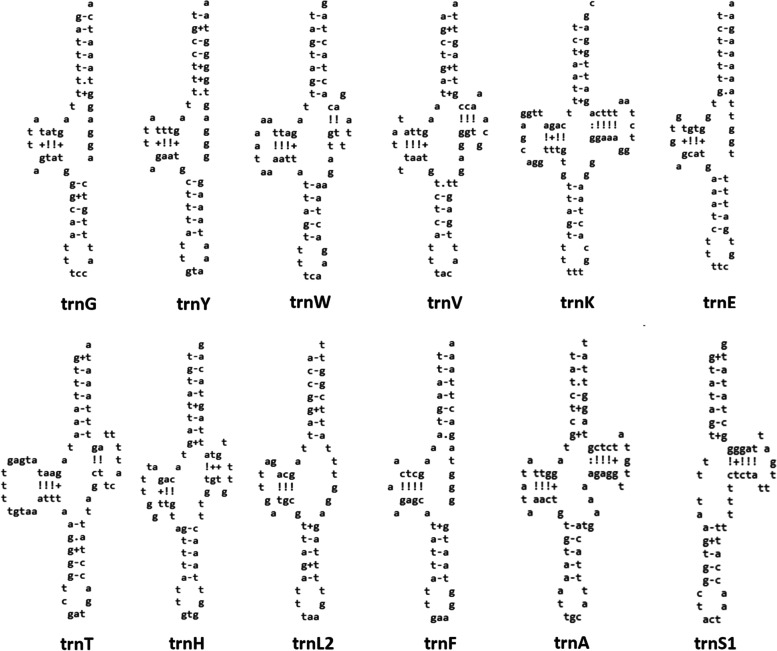
Secondary structures of tRNA genes of *H. pseudobagri* inferred by ARWEN

To the extent to which the automatic annotation was successful, the assembled transcriptomic mitogenome had identical architecture to the DNA mitogenome, but several PCGs were missing (*nad6, nad3, nad4L*, and *nad2*) and many automatically annotated PCGs exhibited deletions in comparison to PCGs in the manually annotated DNA-based mitogenome (Additional file 1: Figure S6). As the two mitogenomic sequences were largely identical, these were artefacts produced by automatic annotation. Deletions were observed at the 5’ end (*cytb, cox2,* and *atp6*), at the 3’ end (*cox1*), or even at both ends (*nad5, nad4, nad1,* and *cox3*). These annotation artefacts aside, all sequences were otherwise identical. The only exception was three different bases at the 5’ end of *cox1*. In addition, *rrnS* was not identified, and only seven tRNA genes were annotated (Table 3). All annotated tRNA genes were identical to the ones from the DNA assembly. Among the tRNAs annotated in the original mitogenome that ARWEN failed to fold into a secondary structure (Fig. 3), only *trnP* was annotated in the transcriptomic assembly.

**Table 3:** tRNA genes in the mitogenome of *H. pseudobagri*. The “folded” column indicates whether the gene could be folded into a cloverleaf-like structure by the ARWEN algorithm. The “missing” column indicates which tRNA structural elements were missing. The “trans” column indicates whether the gene was automatically annotated in the transcriptome

gene	folded	missing	trans	gene	Folded	Missing	trans
trnG	yes	TV-loop	yes	*trnH*	yes		
trnQ	no			*trnL2*	yes	TV-loop	yes
trnY	yes	TV-loop		*trnP*	yes		yes
*trnL1*	no			*trnI*	no		
*trnD*	no			*trnM*	no		
*trnW*	yes		yes	*trnF*	yes	TV-loop	yes
*trnV*	yes			*trnC*	no		
*trnK*	yes			*trnA*	yes		
*trnE*	yes	TV-loop	yes	*trnR*	no		
*trnT*	yes			*trnN*	no		
*trnS2*	no			*trnS1*	yes	D-arm	yes

### tRNA genes

As tRNA genes were very difficult to annotate, we provide an overview of the annotation of tRNA genes in *H. pseudobagri* in Table 3. Additional details are provided in the text. Secondary tRNA structures (where available) are provided in Fig. 3, and screenshots of most alignments and some secondary structures are provided in the Additional file 1: Figures S7-S29.

**Fig. 4:**
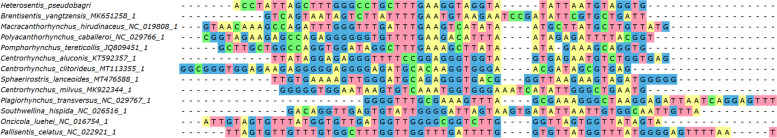
Alignment of *trnS2* genes of Acanthocephala

**Fig. 5:**
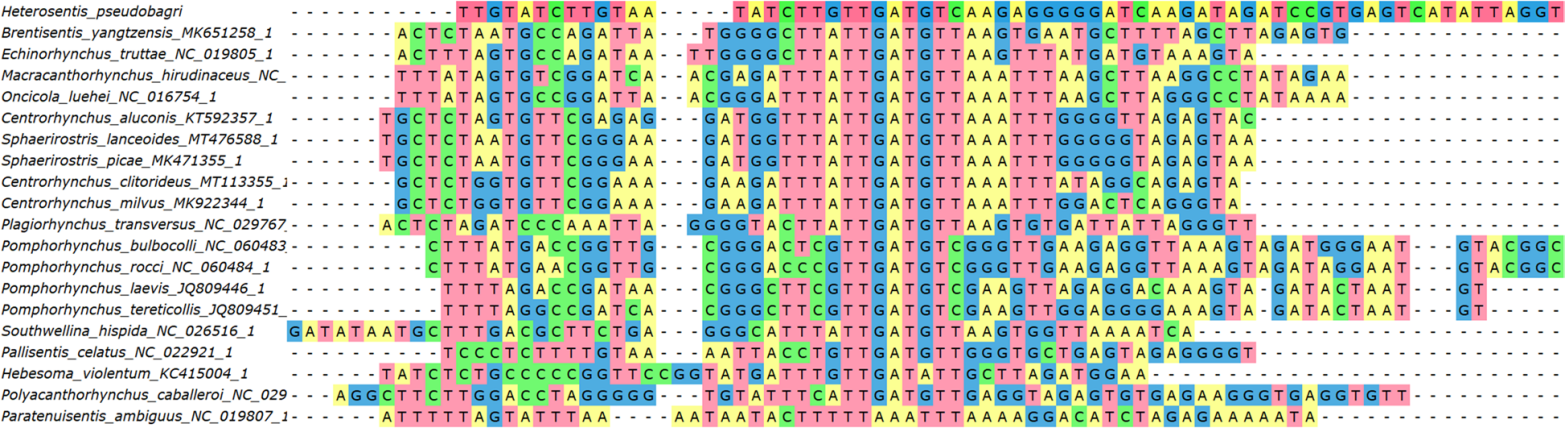
Alignment of *trnI* genes of Acanthocephala

**Fig. 6:**
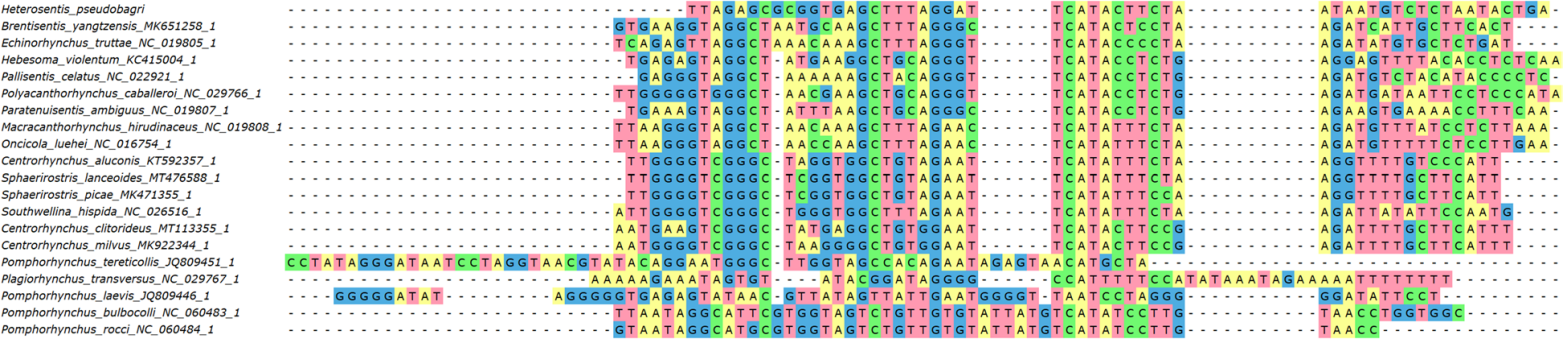
Alignment of *trnM* genes of Acanthocephala

**Fig. 7:**
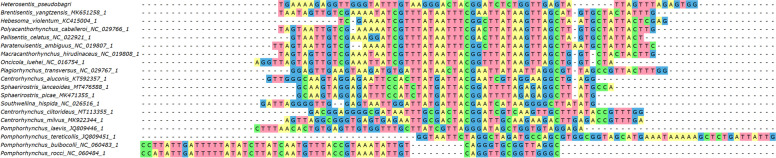
Alignment of *trnR* genes of Acanthocephala

*trnG (tRNA-Gly)*: it was folded into a tRNA by ARWEN in all species apart from *Pallisentis celatus* (NC_022921) and *Pomphorhynchus bulbocolli* (NC_060483). *trnQ (tRNA-Gln)*: as the *cox1-G-Q-Y-rrnL* box is highly conserved in the Acanthocephala (Additional file 1: Figure S5), we attempted to annotate this gene between *trnG* and *trnY*. This allowed us to identify a relatively conserved central segment, putatively comprising the GTC base triplet. ARWEN failed to fold this sequence into a cloverleaf structure. This was also common in the entire dataset: only four species had *trnQ* genes that could be folded. Although the orthologues were highly divergent in the dataset, there was a highly conserved AGATTTTGGGTCTT box in the central segment of the gene, which indirectly suggests that the gene may be functional. *trnY*: it could not be folded in several acanthocephalan species. *trnL1*: the 5’-end and the central part of this gene were highly conserved, but the 3’-end was highly divergent. ARWEN also failed to fold many other orthologues in the dataset. We annotated it approximately, abutting the downstream *nad6*, but it may as well overlap with it by a few bases. *trnD*: after we initially failed to identify this gene, we searched the expected location between *nad6* and *atp6*, conserved in other species. We managed to identify a conserved central part, but 5’ and 3’ ends were poorly conserved, and we failed to fold it into a tRNA structure, so the annotation of ends was approximate. *trnW*: this gene was conserved in most species apart from *Pomphorhynchus rocci* (NC_060484). *trnV*: it was highly conserved in most species in the dataset, and most orthologues used the TAC base triplet. In *C. milvus*, *H. violentum* and *P. celatus*, *trnV* was highly divergent, did not exhibit the conserved central segment with the correct base triplet, and could not be folded into a secondary tRNA structure, so we suspect possible misannotation. *trnK*: we failed to identify this gene in the conserved acanthocephalan V-K-E-T box in *H. pseudobagri*. Instead, the gene was translocated between *nad1* and *rrnS*. It exhibited a relatively conserved central part, including the TTT base triplet, but rather divergent 5’ and 3’ ends. Despite this, it could be folded into a standard cloverleaf structure in *H. pseudobagri*. The gene was highly divergent in several species: *Plagiorhynchus transversus*, *H. violentum, P. celatus, C. milvus,* and *Onicicola luehei*. *trnE*: it was highly divergent in *H. violentum*, and *C. milvus*, where it also did not have the conserved TTC base triplet, so it may be misannotated. *trnT*: we identified this gene in the box between *nad3* and *nad4L*, and ARWEN folded it into a cloverleaf structure, but it recognized it as *trnI*. Given that this is the ancestral position of *trnT*, and that we identified a conserved *trnI* segment in the ancestral acanthocephalan location, it is most likely an artefact. Its 5’ end and central segment comprising the TGT triplet were highly conserved, but the 3’ end was divergent, which may be the underlying reason for the misidentification by ARWEN. The orthologues in *P. caballeroi* and *O. luehei* exhibited almost no homology to other species, but they did exhibit the TGT triplet, so further studies are needed to discern the evolution of this gene in these species. *trnS2*: the position of this gene is not highly conserved in acanthocephalans, but in most species it was encoded between *trnT* and *nad4L*. We found a conserved central segment of this gene comprising the conserved TGA triplet in the above location in *H. pseudobagri*, but 5’ and 3’ ends were highly divergent (Fig. 4). Probably as a result of that, ARWEN failed to fold the putative gene into the cloverleaf structure. Many other species exhibited highly divergent sequences, without the conserved triplet in the expected position. *trnH*: the position of this gene between *nad4* and *nad5* is almost perfectly conserved in Acanthocephala and its sequences, especially the central part comprising the GTG triplet, were also relatively well conserved in most species, including *H. pseudobagri*. *trnL2*: the position of this gene between *nad5* and *trnP* is almost perfectly conserved in the Acanthocephala. Sequence-wise, only the central segment comprising the TAA triplet was highly conserved. *trnP*: the position of this gene between *trnL2* and *cytb* is almost perfectly conserved in the Acanthocephala. Sequence-wise, only the central segment comprising the TGG triplet was highly conserved. *trnI*: At first we failed to annotate *trnI* in *H. pseudobagri*. The segment between *nad1* and *rrnS* is rather divergent in acanthocephalans, but in many species it contains *trnI* and *trnM*, so we searched downstream from *nad1*. We identified a largely conserved central motif comprising the GAT triplet: CTTGTTGATGTCAAG (Fig. 5). Apart from the central segment, 5’ and 3’ ends did not resemble other orthologues and ARWEN failed to fold it, so we annotated the gene only approximately. As regards other mitogenomes in the dataset, ARWEN failed to fold most genes, but the few that were folded had a standard cloverleaf structure. Also, this gene was most probably misannotated in *P. ambiguus*, as it had no resemblance to other orthologues even in the otherwise conserved central segment. *trnM*: initially we failed to annotate this gene within the *H. pseudobagri* mitogenome. Searching the segment between *nad1* and *rrnS* and aligning the remaining orthologues allowed us to identify the conserved TCATA motif (comprising the CAT triplet) (Fig. 6). On this basis, we putatively identified this gene, but due to the absence of almost any similarity with orthologues aside from the central segment, the annotation of this gene was merely approximate. This gene was highly divergent in *Pomphorhynchus*: some of them exhibited the conserved CAT triplet, but others did not, so it may be that some were misidentified. *trnF*: the location and the sequence of *trnF* were conserved in most species. Notably, some of the species in the dataset possessed *trnF* genes for which ARWEN indicated that they may simultaneously code for other tRNA genes; for example *trnE* (TTC) in *P. laevis* (JQ809446). *trnC*: the location, the sequence, and the base triplet (GCA) of *trnC* were conserved in most species. ARWEN failed to fold the tRNA of *H. pseudobagri*, but most orthologues had unusual structures, and several orthologues could be folded into two different tRNAs (*trnA* in *C. milvus*, and *P. transversus*, *trnA* and *trnL* in *P. ambiguus*). *trnA*: the location, the sequence, and the base triplet (TGC) of *trnA* were conserved in most species. *trnR*: at first we did not manage to annotate this gene in the mitogenome of *H. pseudobagri*, and ARWEN failed to fold many of the orthologues in the dataset. Among those that were successfully folded, the DNA base triplets used within the dataset were: TCG and ACG. We searched for these triplets in the putative ancestral position, where the gene is found in most other species: between *trnA* and *trnN*. This allowed us to identify a relatively conserved central fragment (GACTACGGATC) in *H. pseudobagri* (Fig. 7). The 5’ and 3’ ends exhibited a low level of conservation, so they were annotated approximately, and ARWEN failed to fold it into a tRNA structure. In *Brentisentis yangtzensis*, *Polyacanthorhynchus caballeroi*, and *Macracanthorhynchus hirudinaceus* orthologues, ARWEN identified two different tRNAs: *trnR* (ACG or TCG) and *trnT* (TGT). Two *Pomphorhynchus* species (*tereticolis*, and *laevis*) exhibited highly divergent sequences, bearing almost no similarity to other orthologues. *trnN*: this gene was also identified only on the basis of its ancestral position (between *trnR* and *trnS1*) and the conserved central base triplet (GTT). The 5’ and 3’ ends exhibited a low level of conservation and ARWEN failed to fold it into a tRNA structure. However, ARWEN also failed to fold most other sequences in the dataset. *trnS1*: similar to the previous two genes, this gene was also identified only on the basis of its ancestral position (between *trnN* and *nad2*) and the conserved base triplet ACT. The 5’ and 3’ ends exhibited a low level of conservation.

## Discussion

We sequenced the first mitogenome for the family Arhythmacanthidae. The topology inferred using mitogenomic data mostly, but not completely, corresponded to previous mitogenomic studies, most notably in rendering Echinorhynchida paraphyletic [17–19, 33, 34]. The paraphyly of this order was also observed in some nuclear molecular data-based studies [9, 35]. A previous mitogenomic study recognised Illiosentidae and Rhadinohynchidae as lineages that cause topological instability within the Palaeacanthocephala [19]. Three different topologies were previously observed concerning the basal (sister-lineage to all other members) radiation of Palaeacanthocephala: 1. The sister-clade of Illiosentidae + Rhadinohynchidae [17, 18, 34], 2. Illiosentidae [19], and 3. Rhadinohynchidae [19]. Topology 1 was obtained in our study, but there are some indications that this may be a long-branch attraction artefact [19], so more data are needed to resolve this question. The IQ-TREE analysis also found that all but one sequence failed the composition chi^2^ test (p-value < 5%; df = 3), which is indicative of strong compositional heterogeneity in the dataset, and supports previous findings [1, 19, 36]. Compositional heterogeneity violates the assumptions of many phylogenetic algorithms and it may produce homoplastic base composition patterns in distant lineages, causing artefactual clustering, so mitogenomic data may produce misleading findings when used to conduct phylogenetic analyses of the entire acanthocephalan dataset [37–39]. As the phylogeny of Acanthocephala remains debated, largely due to the absence of suitable molecular data, this prevents us from making any conclusions about the phylogeny of Arhythmacanthidae and Acanthocephala. Future studies should strive to sequence mitogenomes of unrepresented lineages and make sure that different algorithms and datasets produce congruent topologies.

The newly sequenced mitogenome of *H. pseudobagri* possessed several features common to Acanthocephala. Mitogenomic architecture with all genes encoded on the same strand is common not only in Acanthocephala but also in Gnathifera [24, 29]. The average mitogenome size is relatively small in Syndermata [29], and the mitogenome of *H. pseudobagri* exhibited several signatures of selection for small size, including relatively small rRNA genes in comparison to most other animals [29]. The newly sequenced *H. pseudobagri* exhibited a unique gene order, but it diverged from other acanthocephalan mitogenomes only in terms of tRNA gene arrangement. We annotated only 12 PCGs, as *atp8* appears to be absent from all sequenced acanthocephalan mitogenomes [24, 29], although it should be noted that identification of *atp8* can be very difficult in some lineages [40]. In addition to the apparent absence of *atp8*, the annotation of several other PCGs in this mitogenome was very difficult due to them being highly divergent (*nad6, nad4L*…). This observation was further supported by the low efficiency and precision of automatic annotation conducted using the transcriptomic data assembly. This is indicative of the fast evolution of some PCGs, which in turn suggests relaxed purifying selection pressures or high levels of directional evolution. As several studies found evidence that parasitism might be associated with elevated mitochondrial sequence evolution rates [41–46], the parasitic life history of acanthocephalans may be an important variable underlying this rapid evolution.

Annotation of tRNA genes is also often challenging due to the sporadic degeneration of their typical secondary structure in some lineages [47], tRNA remoulding [48] and incomplete tRNA sets in some lineages [23, 25–28]. Indeed, in acanthocephalans, tRNAs commonly lack either the TWC arm or the DHU arm, or even both [15, 29–31, 33]. Our initial searches managed to identify only seven tRNAs in the mitogenome of *H. pseudobagri*. As previous studies did not report large numbers of missing tRNA genes in acanthocephalan mitogenomes, we attempted to identify these genes manually on the basis of their location in other acanthocephalan species. We only succeeded to annotate all tRNA genes by focusing only on identifying the conserved central section comprising the DNA base triplet (anti-codon), but ignoring the 5’ and 3’ ends, as well as the secondary structure. In many of these tRNAs, 5’ and 3’ ends did not exhibit any similarity to the available orthologues, and ARWEN failed to fold them into a tRNA molecule. There are multiple possible explanations for this phenomenon. Incomplete tRNA sets have been observed in multiple animal lineages, and they are believed to be compensated for by the import of tRNAs from the cytoplasm [47]. We should therefore consider the possibility that these highly divergent tRNA genes have lost their functionality (i.e. became pseudogenes). If an entire gene undergoes pseudogenisation, purifying selection stops acting on its entire sequence, so the entire sequence should evolve at a similar rate (accounting for minor differences in mutational pressures). However, this is not what we observed in the mitogenome of *H. pseudobagri*. We observed a highly conserved central segment and rapidly evolving flanking sequences. This indicates that flanking segments evolve under a relaxed purifying selection, whereas central segments evolve under a strong purifying selection. This is in disagreement with the hypothesis of nonfunctional pseudogenes, so we should look for alternative explanations.

Truncated tRNA genes that undergo extensive posttranscriptional editing were reported in Onychophora [49]. It is also possible that tRNA genes in the mitogenome of *H. pseudobagri* are truncated. We annotated these genes manually by extending them in both directions to correspond to other orthologues lengthwise. This method generated many large overlaps; e.g. 33 bp between *trnP* and *cytb*, 26 bp between *nad5* and *trnL2*, 25 bp between *trnC* and *cox3*, 20 bp between *nad3* and *trnW*, etc. Even if we account for the possibility of annotation errors, this would be considered unusual in most metazoan lineages [47]. Furthermore, overlaps are most commonly found between two tRNA genes encoded on different strands [47], whereas the above-described overlaps in *H. pseudobagri* are largely between a tRNA gene and a PCG encoded on the same strand. Exceptionally large overlaps, and even tRNA genes fully integrated into neighbouring genes, have been observed in isopods (Arthropoda) [50]. These tRNAs also undergo extensive posttranscriptional tRNA processing which restores them to more conventional structures [50]. Some basic tRNA editing mechanisms and associated machinery exist in many animal lineages [49, 51, 52]. On this basis, we hypothesise that (some) tRNA genes in (some) acanthocephalans may also undergo posttranscriptional processing. We attempted to sequence the transcriptome to test this hypothesis, but unfortunately, standard RNA-seq sequencing methods often cannot be applied efficiently to tRNA genes [53], so transcriptome data failed to produce meaningful findings in this respect.Although most of the cases of tRNA editing in animals appear to be recent evolutionary acquisitions that arose independently in various lineages [51], in most known cases relatively few (1 to 3) nucleotides are edited in tRNA sequences [49]. As tRNA genes of *H. pseudobagri* appear to require substantial editing, we deemed it unlikely that this complex mechanism would evolve independently in *H. pseudobagri*, so we also checked whether genes of other species could be folded using ARWEN. We found evidence that highly divergent tRNAs that cannot be folded into a cloverleaf structure are found in many acanthocephalan species. Remarkably, in many cases, tRNA genes from the family Pomphorhynchidae strongly differed from other genes in the dataset. Furthermore, the tRNAs of Archiacanthocephala and Eoacanthocephala were highly divergent from Palaeacanthocephala. This indicates that the putative tRNA editing mechanism is not limited to *H. pseudobagri* and that tRNA genes are evolving rather rapidly in acanthocephalans in general. However, this needs to be confirmed by further studies, as we suspect that the unrecognised existence of unusual tRNA genes in acanthocephalans may have resulted in some annotation artefacts in previously sequenced mitogenomes. Notably, extensive tRNA editing was previously associated with relaxed purifying selection pressures [49], which is in agreement with our observation that mitochondrial genes in acanthocephalans appear to be evolving at high rates. As our study did not produce empirical evidence for tRNA editing, this explanation for non-standard tRNA genes in acanthocephalan mitogenomes remains hypothetical. It is necessary to sequence more acanthocephalan mitogenomes, especially focusing on unrepresented lineages, and experimentally explore the unusual features of tRNA evolution in this lineage.

## Methods

### Samples

The parasite used for DNA extraction and mitogenome sequencing was collected on 07/09/2018 from the intestinal tract of the host, yellow catfish *Pelteobagrus fulvidraco*, sampled from the South Dongting Lake, Yuanjiang county, Hunan province, China (28°50’N, 112°23’E). The host fish was euthanised using 250 mg/L MS-222 buffered with sodium bicarbonate for a pH between 7–7.5. Since there are no molecular data available for this species, we identified the parasite as *Heterosentis pseudobagri* (Wang & Zhang, 1987) on the basis of its morphology and host according to previous descriptions [8, 54]. The parasite used for transcriptome sequencing was collected on 11/06/2022 from the same host species caught in the eastern part of the same Dongting Lake and identified in the same way. Both sampled specimens were washed and blotted dry alive. The specimen used for DNA extraction was then stored in absolute ethanol, whereas the one used for RNA extraction was flash-frozen alive in liquid nitrogen.

### Mitogenome sequencing, annotation and assembly

The total DNA was isolated from a single specimen and used as the PCR template. Primers were designed to produce amplicons overlapping by approximately 100 bp. PCR products were sequenced bidirectionally by the Bio-Transduction Lab in Wuhan using the Sanger method and the same set of primers that were used for the amplification (Additional file 1: Table S1). Sequenced data were quality-inspected using sequencing chromatograms; 20–30 bases at both ends were deleted from each amplicon. The mitogenome was assembled manually using DNAstar v7.1 [55], ensuring that overlaps between fragments were identical. PCGs and rRNAs were approximately located using DNAstar and MITOS [56] and then manually fine-tuned according to the orthologous sequences using BLAST and BLASTx [57] and PhyloSuite [58, 59]. tRNAs were annotated using MITOS [56] and ARWEN [60] tools, but some tRNAs had to be identified manually via comparisons with related species. The secondary structure of tRNAs was further studied using ARWEN. Tools such as tRNAscan, which searches for a complete cloverleaf structure, are not suitable for the identification of tRNAs that exhibit nonstandard secondary structures. ARWEN was designed especially for this purpose: it first identifies only the most conserved domain, the anticodon stem, and subsequently searches for the presence of D-stem and T-stem structures, and the search for an acceptor stem then provides specificity. Due to this high sensitivity, ARWEN is also prone to producing a substantial false discovery rate [47]. The assembled circular mitogenome was visualised using OGDRAW [61].

### Transcriptome sequencing and mitogenome assembly from transcriptomic data

The total RNA was extracted using Qiagen's animal tissue RNA extraction kit. oligodT magnetic beads were used to separate mRNA from other RNAs by binding to its poly(A). First-strand cDNA was synthesized using random hexamer primer and reverse transcriptase RNase H, and second-strand cDNA synthesis was subsequently performed using DNA Polymerase I and RNase H. The remaining overhangs were converted into blunt ends via exonuclease/polymerase activities, 3’ ends of DNA fragments were adenylated, DNBSEQ-T7 sequencer-specific adapters were added, and a cDNA library was constructed by PCR enrichment. Then, the cDNA library and the probe (single-stranded DNA labelled with biotin) were hybridized in a liquid phase. After the incubation, streptomycin-labeled magnetic beads were added. After the elution of non-target sequences and other impurities to obtain a target region library, the products were enriched by PCR. Finally, the double-stranded target region library was denatured, circularized and digested to obtain single-stranded circular DNA, which was then subjected to Rolling Circle Amplification to obtain the amplified product, DNA Nano Ball. After the library was constructed, Qubit was used for quantitative quality control. The prepared DNB was loaded on a microarray chip (Patterned Array), and the combined probe-anchored polymerization technology (cPAS, Combinatorial Probe-Anchor Synthesis) was used for sequencing. After the polymerization reaction on nanospheres, a high-resolution imaging system was used to collect, read and identify the light signal to obtain the single base sequence information, and then proceed to the next cycle to obtain the next base sequence information, and finally obtain the information after multiple cycles. MITGARD was used to assemble the mitogenome from transcriptomic data [62], and mitoZ [63] was used to annotate it.

### Comparative and phylogenetic analyses

For the mitogenomic dataset, we used almost all available annotated Acanthocephala mitogenomes (9/4/2022), aside from some conspecific sequences. Two Rotifera mitogenomes were used as outgroups: *Rotaria rotatoria* (Bdelloidea: Rotifera: Syndermata) [31] and *Philodina citrina* [15]. PhyloSuite [58, 59] was used to retrieve all mitogenomes, standardise annotation, retrieve detailed taxonomic info from the NCBI, extract mitogenomic data, and generate comparative tables. GC skew was calculated by PhyloSuite using the following formula: (G-C)/(G + C). For the NCR visualization, the threshold was set to 100 bp in PhyloSuite. For the phylogenetic analyses, nucleotide sequences of all 12 PCGs were aligned in the codon mode using the accurate E-INS-i strategy in MAFFT [64] and alignments were then refined using MACSE [65]. Aligned genes were concatenated using PhyloSuite, and the optimal evolutionary model and partitioning scheme were inferred using ModelFinder [66]: partition 1—*ap6, cox3, nad2, nad3* (model = TVM + F + R4); partition 2—*cox1* (TVM + F + I + G4); partition 3 – *cox2, cytb, nad1* (GTR + F + I + G4); and partition 4—*nad4L, nad4, nad5, nad6* (GTR + F + R4). Phylogenetic analysis was conducted using IQ-TREE [67] with 20,000 ultrafast bootstraps [68] and an SH-aLRT test (1,000 replicates) [69]. By default, IQ-TREE uses a composition chi-square test to assess the homogeneity of character composition for every sequence in the alignment. For the three single-gene datasets (*cox1*, *18S*, *28S*) we downloaded all available Acanthocephala homologues, pruned all sequences that were too short or misaligned, and left only one or two sequences per species, apart from the Arhythmacanthidae, for which we left all sequences which could be aligned. The final datasets comprised 96 *18S* sequences (1 Arhythmacanthidae), 174 *28S* sequences (2 Arhythmacanthidae), and 157 *cox1* sequences (4 Arhythmacanthidae). Phylogenetic analyses were conducted as described above, except for the fact that sequences were aligned only using MAFFT (not in the codon mode) and that we used the GTR + G + I evolutionary model in all three analyses. There is evidence that using GTR + G + I, which is the most parameter-rich model, allows skipping the evolutionary model selection step in phylogenetic analyses without producing any detrimental effects on the accuracy of topology [70]. iTOL [71] was used to visualise the phylogeny and architecture with files generated by PhyloSuite. MEGA X [72] and Unipro UGENE 44.0 [73] were used to visualise tRNA alignments. Some default NCBI taxonomic identity was manually changed according to the more often updated WORMS database (e.g. the class attribution of *Polyacanthorhynchus caballeroi* was changed from Polyacanthocephala to Eoacanthocephala).

## Supplementary Information

**Additional file 1: Additional figures and tables.** **Figure S1.** Maximum Likelihood phylogenetic analysis of the Acanthocephala mitogenomic dataset based on nucleotide sequences of all 12 mitogenomic protein-coding genes. Numbers at nodes indicate SH-aLRT support, H. pseudobagri is highlighted using colour shading, and family, order and class-level identities are shown to the right in that order (left to right). **Figure S2. **Bayesian Inference phylogenetic analysis of the Acanthocephala mitogenomic dataset based on nucleotide sequences of all 12 mitogenomic protein-coding genes.Numbers at nodes indicate posterior probability support, *H. pseudobagri *is highlighted using colour shading, and family, order and class-level identities are shown to the right in that order (left to right). **Figure S3. **Maximum Likelihood phylogenetic analysis of the Acanthocephala mitogenomic dataset based on 1st and 2nd codon positions of nucleotide sequences of all 12 mitogenomic protein-coding genes.Numbers at nodes indicate SH-aLRT support, *H. pseudobagri *is highlighted using colour shading, and family, order and class-level identities are shown to the right in that order (left to right). **Figure S4.** The architecture of the mitogenome of *H. pseudobagri*. **Figure S5. **Gene orders of *H. pseudobagri *and the remaining available Acanthocephala mitogenomes.Species names are given with corresponding GenBank accession numbers. Family, order, and class-level taxonomic identities are shown to the right. NCR indicates an intergenic region >100 bp. **Figure S6. **The mitogenome of *H. pseudobagri *assembled using the transcriptome data. **Figure S7.** Acanthocephalan trnQ alignment. **Figure S8. **Acanthocephalan *trnG *(tRNA-Gly) alignment. **Figure S9. **Acanthocephalan trnY alignment. **Figure S10. **Acanthocephalan *trnL1 *alignment. **Figure S11. **Acanthocephalan *trnD *alignment. **Figure S12. **Acanthocephalan *trnW *alignment. **Figure S13. **The alternative folding of *Heterosentis pseudobagri trnW *inferred by ARWEN (*trnS*). **Figure S14. **Acanthocephalan *trnV *alignment. **Figure S15. **Acanthocephalan *trnK *alignment. **Figure S16. **Acanthocephalan *trnE *alignment. **Figure S17. **Acanthocephalan *trnT *alignment. **FigureS18. **Acanthocephalan *trnS2 *alignment. **Figure S19. **Acanthocephalan *trnH *alignment. **Figure S20. **Acanthocephalan *trnL2 *alignment. **Figure S21. **Acanthocephalan *trnP *alignment. **Figure S22. **Acanthocephalan *trnI *alignment. **Figure S23. **Acanthocephalan trnM alignment. **Figure S24. **Acanthocephalan trnF alignment. **Figure S25. **Acanthocephalan trnC alignment. **Figure S26. **Acanthocephalan trnA alignment. **Figure S27. **Acanthocephalan trnR alignment. **Figure S28. **Acanthocephalan trnN alignment. **Figure S29. **Acanthocephalan trnS1 alignment. **Table S1. **Primers used for the amplification and sequencing of the mitogenome of *H. pseudobagri*.

**Additional file 2. **Comparative analyses of the mitogenomicacanthocephalan dataset.

## Data Availability

All data generated or analysed during this study are included in this published article, its supplementary information files and the NCBI’s GenBank repository under the following accession numbers: OP278658 (the complete mitogenome; https://www.ncbi.nlm.nih.gov/nuccore/OP278658.1/), OP286594 (*18S*; https://www.ncbi.nlm.nih.gov/nuccore/OP286594.1/), and OP286859 (*28S*; https://www.ncbi.nlm.nih.gov/nuccore/OP286859.1/). Transcriptome data are available from the NCBI’s SRA database under the accession number PRJNA877466. GenBank accession numbers of all mitogenomic sequences used in the analyses are available in Additional file 2.

## Abbreviations

bp: Base pair
NCR: Non-coding region
PCG: Protein-coding gene
