# Supplementary material for: The mitochondrial genome of Heterosentis pseudobagri (Wang & Zhang, 1987) Pichelin & Cribb, 1999 reveals novel aspects of tRNA genes evolution in Acanthocephala
Source: BMC Genomics. 2023 Mar 2;24:95. doi: 10.1186/s12864-023-09177-9 (PMC9979467; doi:10.1186/s12864-023-09177-9)
Supplement: Supplementary file 1 — Additional file 1: Additional figures and tables. Figure S1. Maximum Likelihood phylogenetic analysis of the Acanthocephala mitogenomic dataset based on nucleotide sequences of all 12 mitogenomic protein-coding genes. Numbers at nodes indicate SH-aLRT support, H. pseudobagri is highlighted using colour shading, and family, order and class-level identities are shown to the right in that order (left to right). Figure S2. Bayesian Inference phylogenetic analysis of the Acanthocephala mitogenomic dataset based on nucleotide sequences of all 12 mitogenomic protein-coding genes.Numbers at nodes indicate posterior probability support, H. pseudobagri is highlighted using colour shading, and family, order and class-level identities are shown to the right in that order (left to right). Figure S3. Maximum Likelihood phylogenetic analysis of the Acanthocephala mitogenomic dataset based on 1st and 2nd codon positions of nucleotide sequences of all 12 mitogenomic protein-coding genes.Numbers at nodes indicate SH-aLRT support, H. pseudobagri is highlighted using colour shading, and family, order and class-level identities are shown to the right in that order (left to right). Figure S4. The architecture of the mitogenome of H. pseudobagri. Figure S5. Gene orders of H. pseudobagri and the remaining available Acanthocephala mitogenomes.Species names are given with corresponding GenBank accession numbers. Family, order, and class-level taxonomic identities are shown to the right. NCR indicates an intergenic region >100 bp. Figure S6. The mitogenome of H. pseudobagri assembled using the transcriptome data. Figure S7. Acanthocephalan trnQ alignment. Figure S8. Acanthocephalan trnG (tRNA-Gly) alignment. Figure S9. Acanthocephalan trnY alignment. Figure S10. Acanthocephalan trnL1 alignment. Figure S11. Acanthocephalan trnD alignment. Figure S12. Acanthocephalan trnW alignment. Figure S13. The alternative folding of Heterosentis pseudobagri trnW inferred by ARWEN (trnS). Figure S14. Acanth [file 12864_2023_9177_MOESM1_ESM.pdf]

Additional file 1

for

The mitochondrial genome of *Heterosentis pseudobagri* (Wang & Zhang, 1987) Pichelin & Cribb, 1999 reveals novel aspects of tRNA genes evolution in Acanthocephala

Jin-Wei Gao, Xi-Ping Yuan, Ivan Jakovlić, Hao Wu, Chuan-Yu Xiang, Min Xie, Rui Song, Zhong-Gui Xie, Yuan-An Wu, Dong-Sheng Ou

Correspondence: ryain1983@163.com

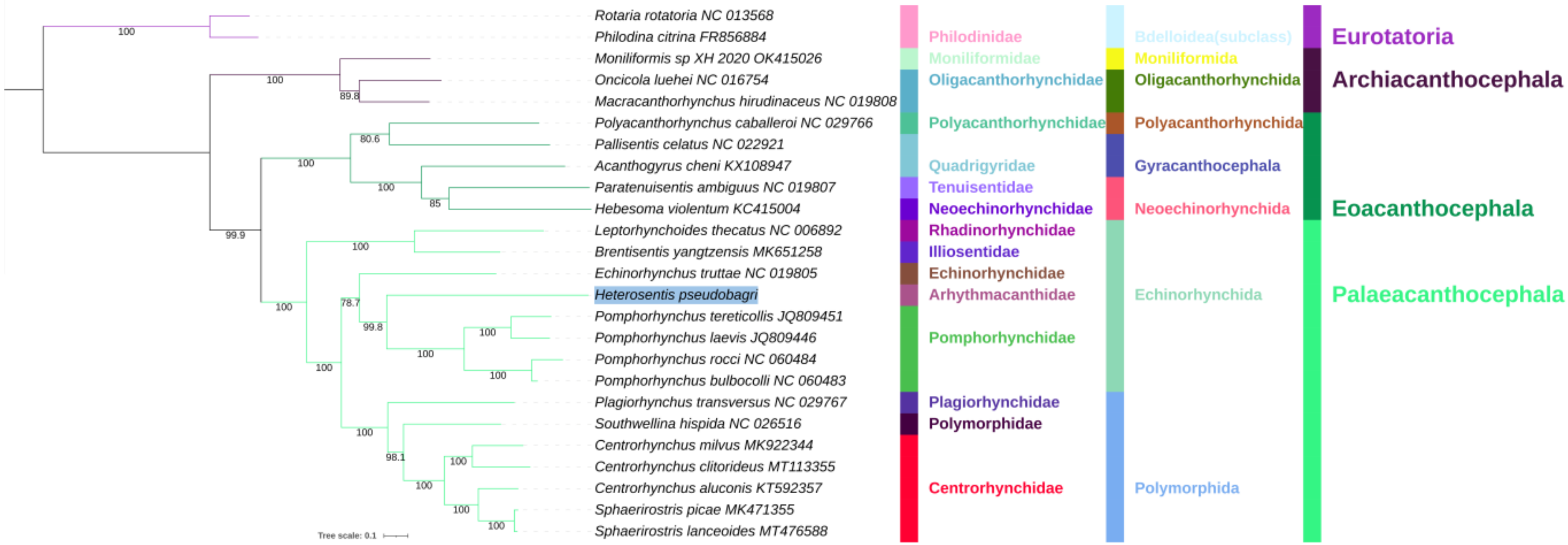

Figure S1. Maximum Likelihood phylogenetic analysis of the Acanthocephala mitogenomic dataset based on nucleotide sequences of all 12 mitogenomic protein-coding genes. Numbers at nodes indicate SH-aLRT support, *H. pseudobagri* is highlighted using colour shading, and family, order and class-level identities are shown to the right in that order (left to right).

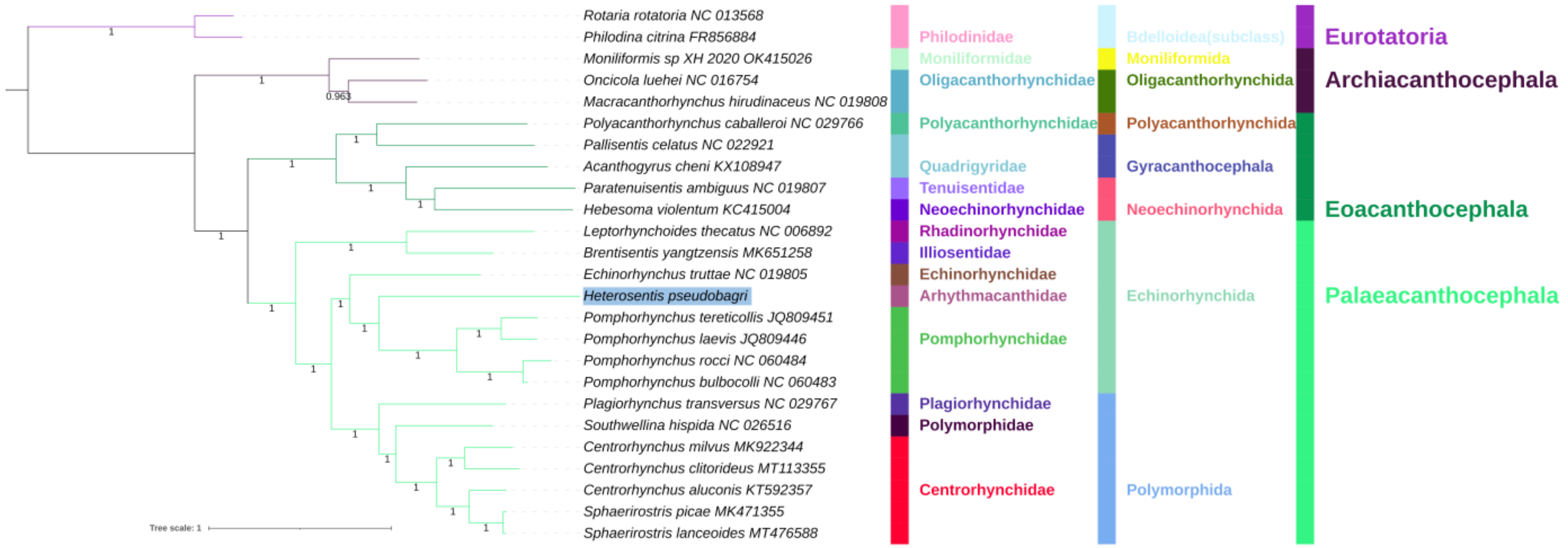

Figure S2. Bayesian Inference phylogenetic analysis of the Acanthocephala mitogenomic dataset based on nucleotide sequences of all 12 mitogenomic protein-coding genes. Numbers at nodes indicate posterior probability support, *H. pseudobagri* is highlighted using colour shading, and family, order and class-level identities are shown to the right in that order (left to right).

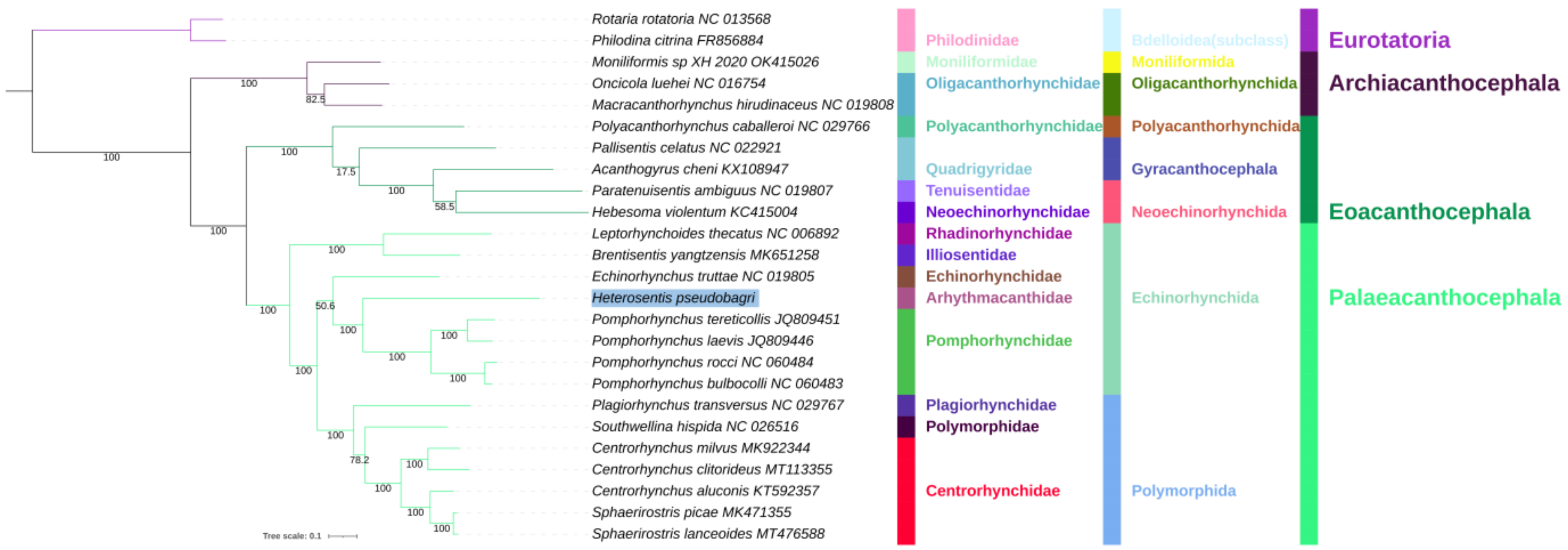

Figure S3. Maximum Likelihood phylogenetic analysis of the Acanthocephala mitogenomic dataset based on 1st and 2nd codon positions of nucleotide sequences of all 12 mitogenomic protein-coding genes. Numbers at nodes indicate SH-aLRT support, *H. pseudobagri* is highlighted using colour shading, and family, order and class-level identities are shown to the right in that order (left to right).

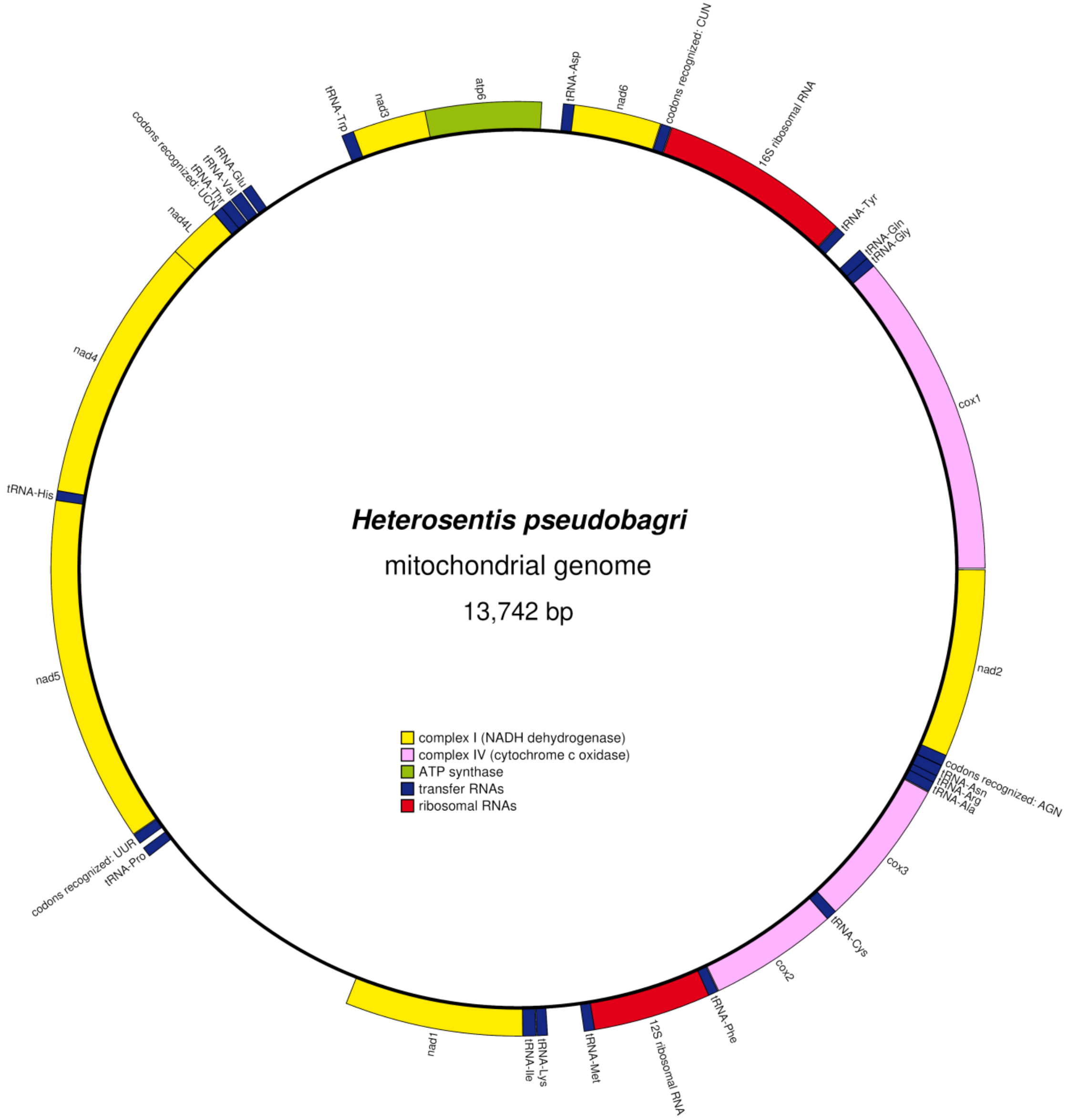

Heterosentis pseudobagri
mitochondrial genome
13,742 bp
complex I (NADH dehydrogenase)
complex IV (cytochrome c oxidase)
ATP synthase
transfer RNAs
ribosomal RNAs
tRNA-Asp
nad6
codons recognized: CUN
16S ribosomal RNA
tRNA-Tyr
tRNA-Gln
tRNA-Gly
cox1
nad2
codons recognized: AGN
tRNA-Asn
tRNA-Arg
tRNA-Ala
cox3
tRNA-Cys
cox2
tRNA-Phe
12S ribosomal RNA
tRNA-Met
tRNA-Lys
tRNA-Ile
nad1
codons recognized: UUR
tRNA-Pro
nad5
tRNA-His
nad4
nad4L
codons recognized: UCN
tRNA-Thr
tRNA-Val
tRNA-Glu
tRNA-Trp
nad3
atp6

Figure S4. The architecture of the mitogenome of *H. pseudobagri*.

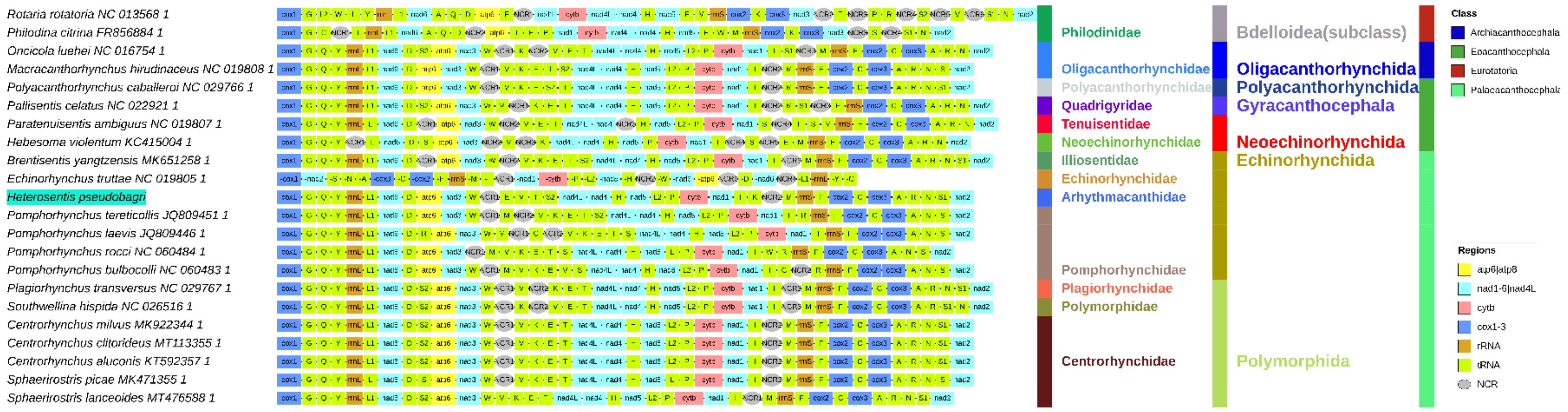

Figure S5. Gene orders of *H. pseudobagri* and the remaining available Acanthocephala mitogenomes. Species names are given with corresponding GenBank accession numbers. Family, order, and class-level taxonomic identities are shown to the right. NCR indicates an intergenic region >100 bp.

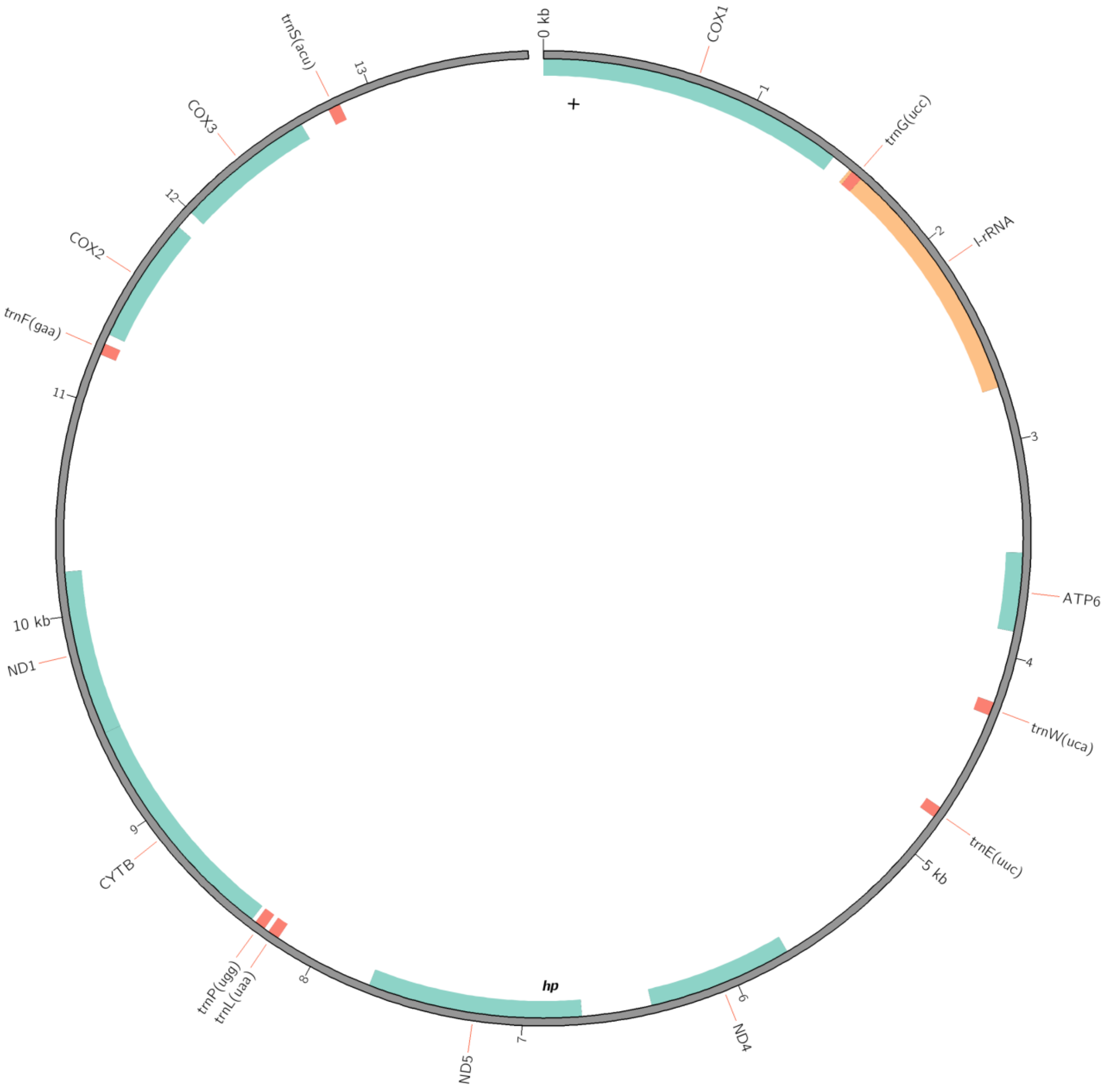

0 kb
COX1
1
trnG(ucc)
2
l-rRNA
3
ATP6
4
trnW(uca)
trnE(uuc)
5 kb
6
ND4
hp
7
ND5
8
trnP(ugg)
trnL(uaa)
9
CYTB
ND1
10 kb
11
trnF(gaa)
COX2
12
COX3
trnS(acu)
13
+

Figure S6. The mitogenome of *H. pseudobagri* assembled using the transcriptome data.

tRNAs

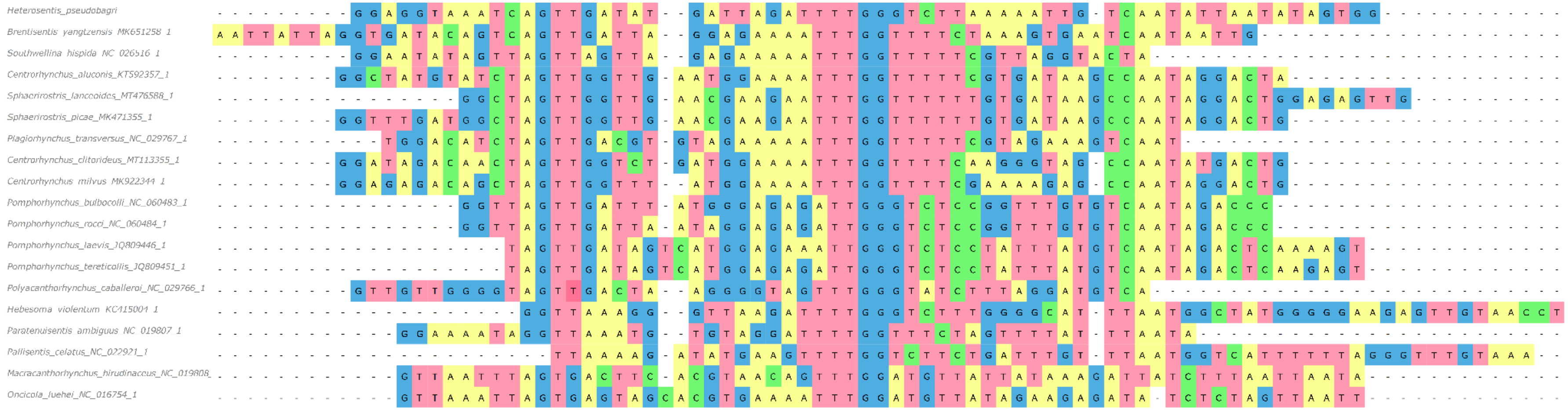

Figure S7. Acanthocephalan trnQ alignment.

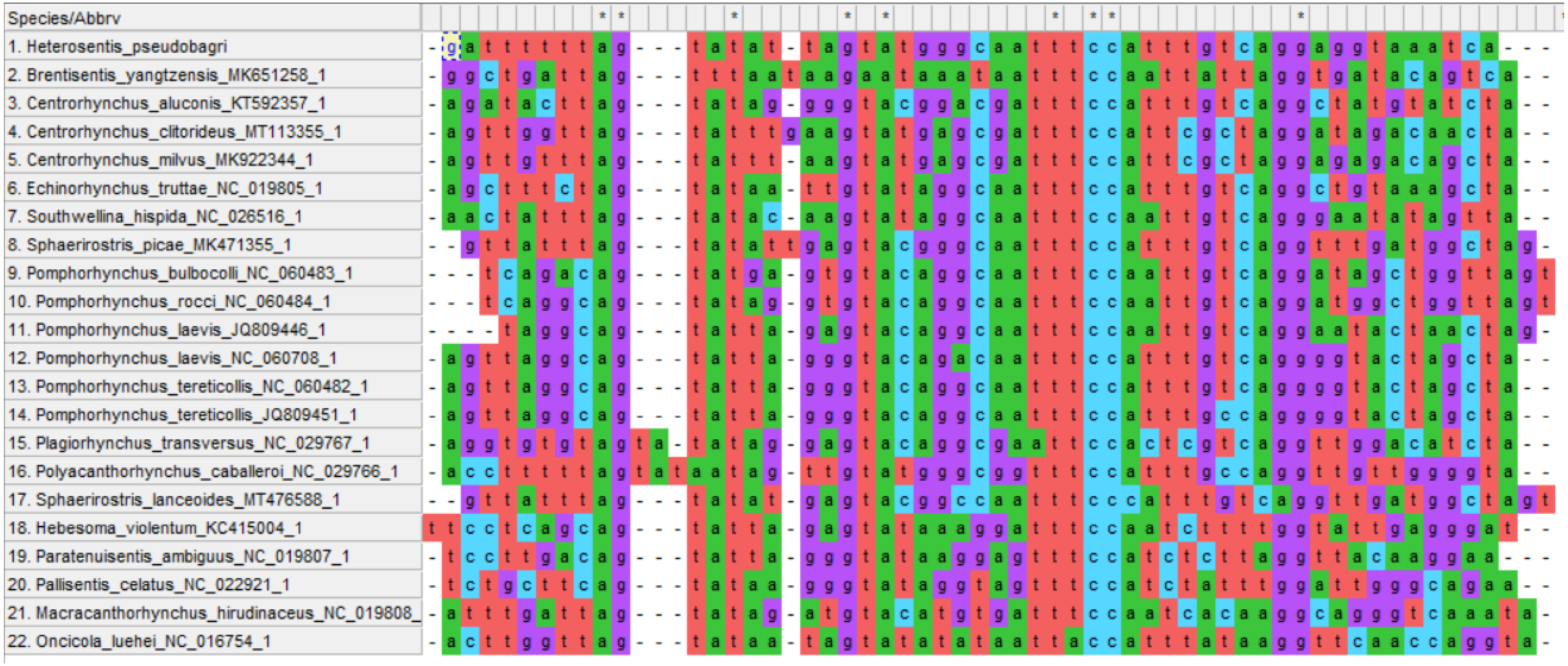

Figure S8. Acanthocephalan *trnG* (tRNA-Gly) alignment.

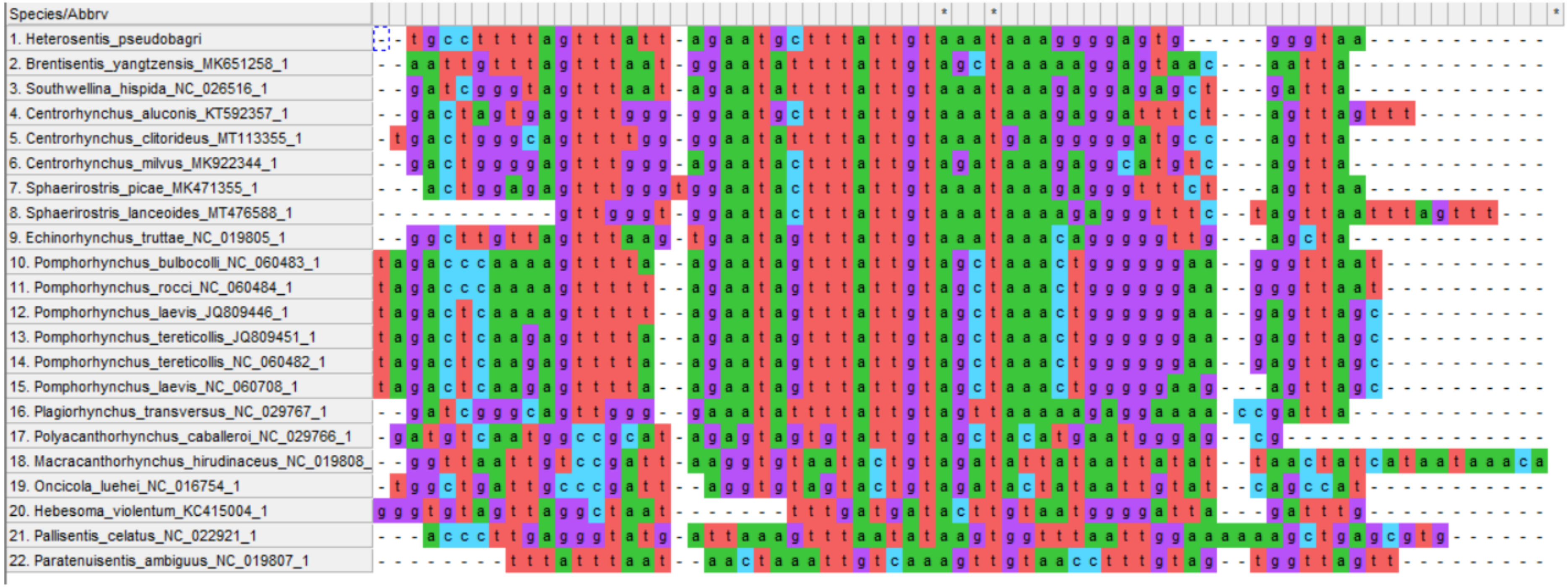

Figure S9. Acanthocephalan trnY alignment.

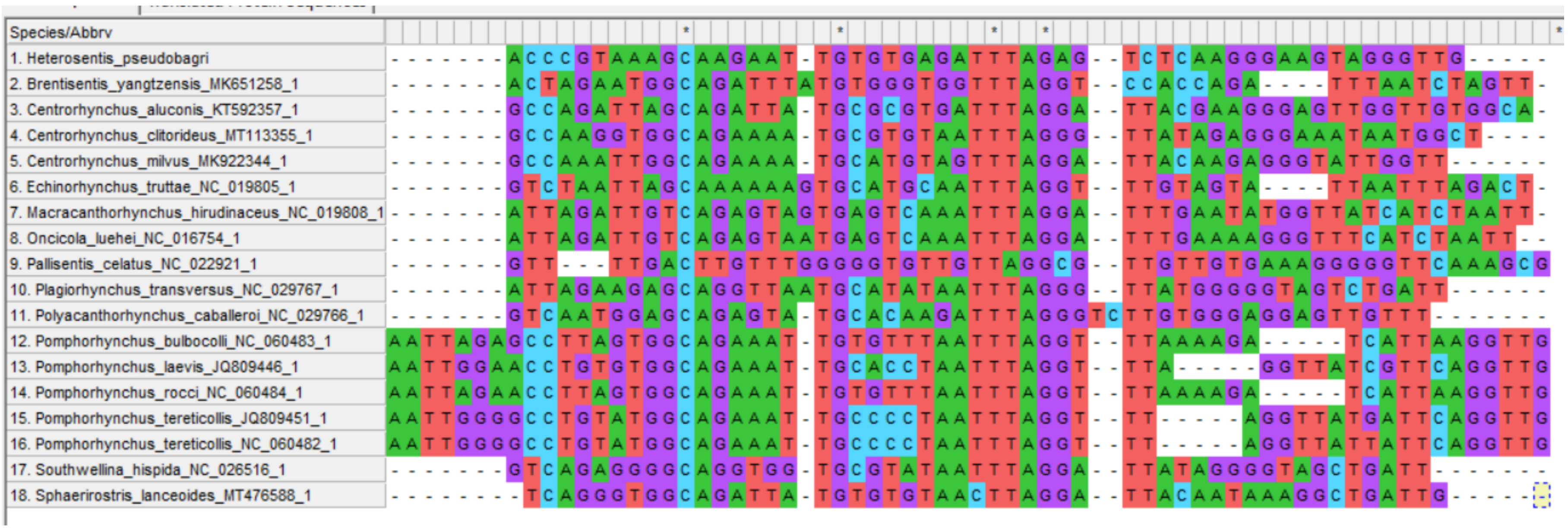

Figure S10. Acanthocephalan *trnL1* alignment.

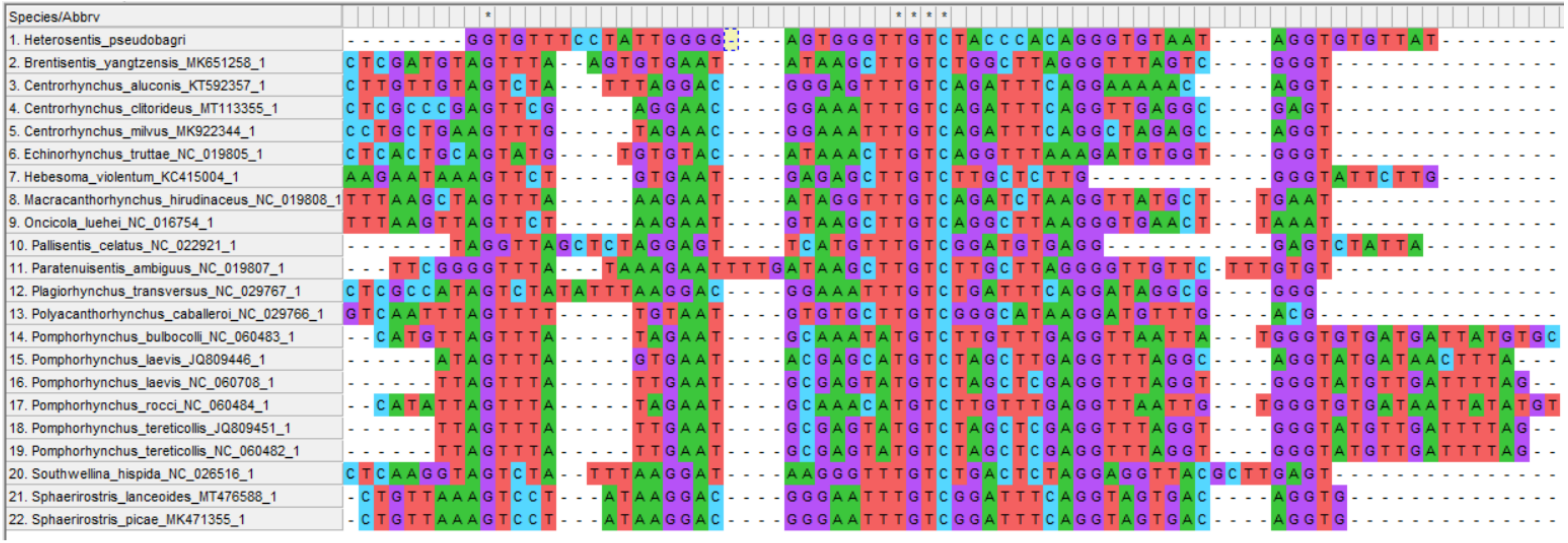

Figure S11. Acanthocephalan *trnD* alignment.

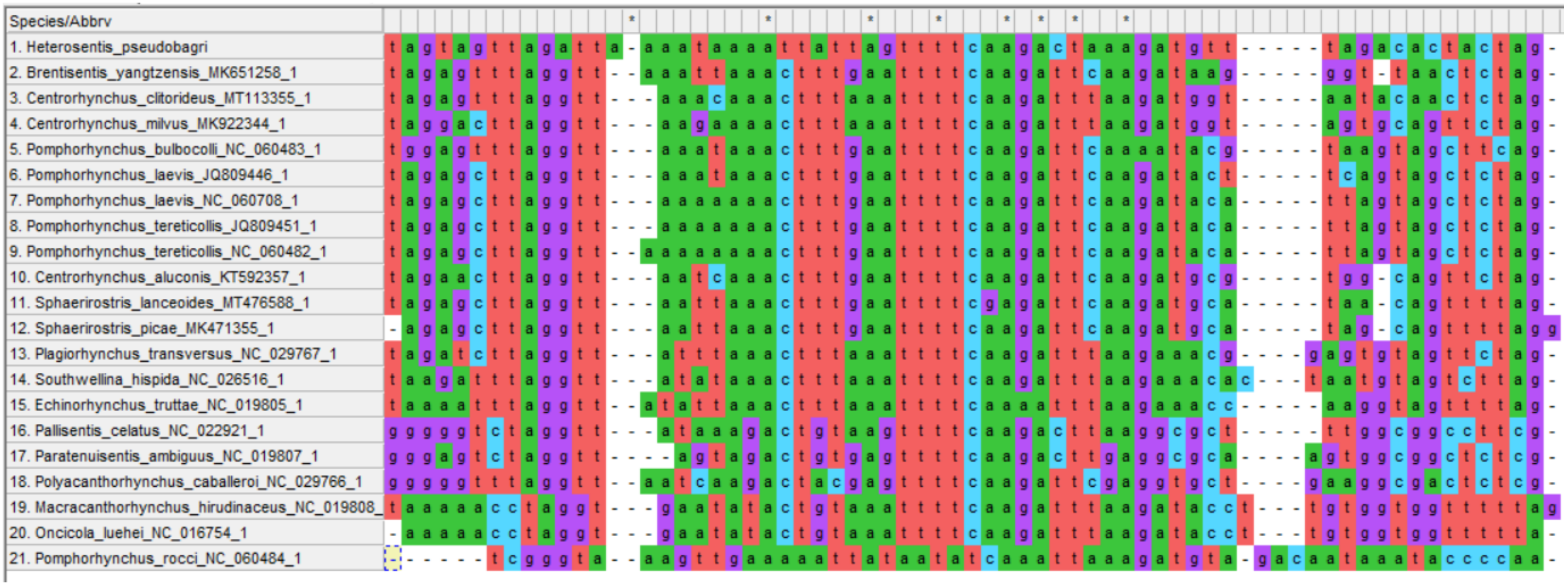

Figure S12. Acanthocephalan *trnW* alignment.

```
               g
             a-t
             c-g
             t-a
             a-t
             c-g
             t-a
             a-t
             g-c       atc
            t   atcatca   t
      c    a    !: !! !   a
     t tgtg     ttttatt  a
     a !!!+     a      tt
       acat    a
      a   c    t
           tt-a
            t-a
            a-t
            g-c
            t-a
           c   a
           t   a
            tga


    mtRNA-Ser(tga)
    75 bases, %GC = 25.3
    Sequence c[54,4]


Overlap with 1: mtRNA-(Stop|Trp)(tca) [1,62]


Number of tRNA genes = 2
Number of TV replacement loop tRNA genes = 0
Number of D replacement loop tRNA genes = 0
tRNA GC range = 22.6% to 25.3%
```

Figure S13. The alternative folding of *Heterosentis pseudobagri trnW* inferred by ARWEN (*trnS*).

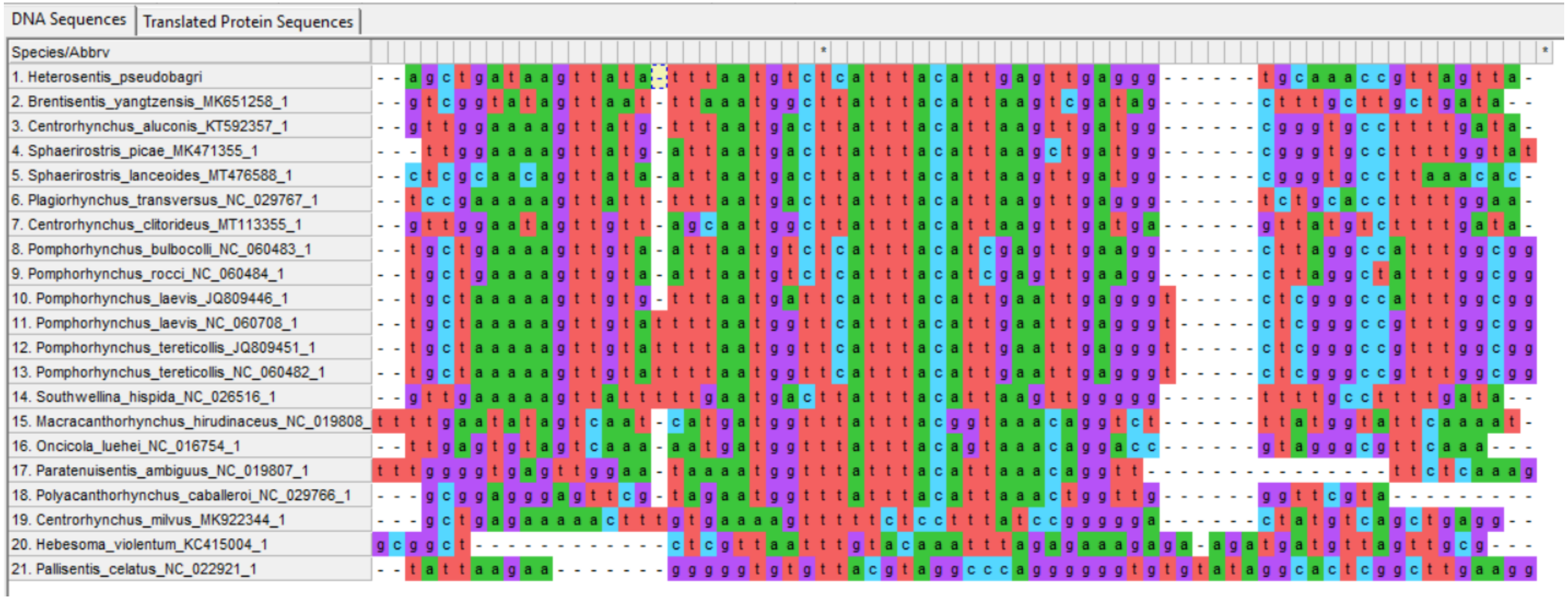

Figure S14. Acanthocephalan *trnV* alignment.

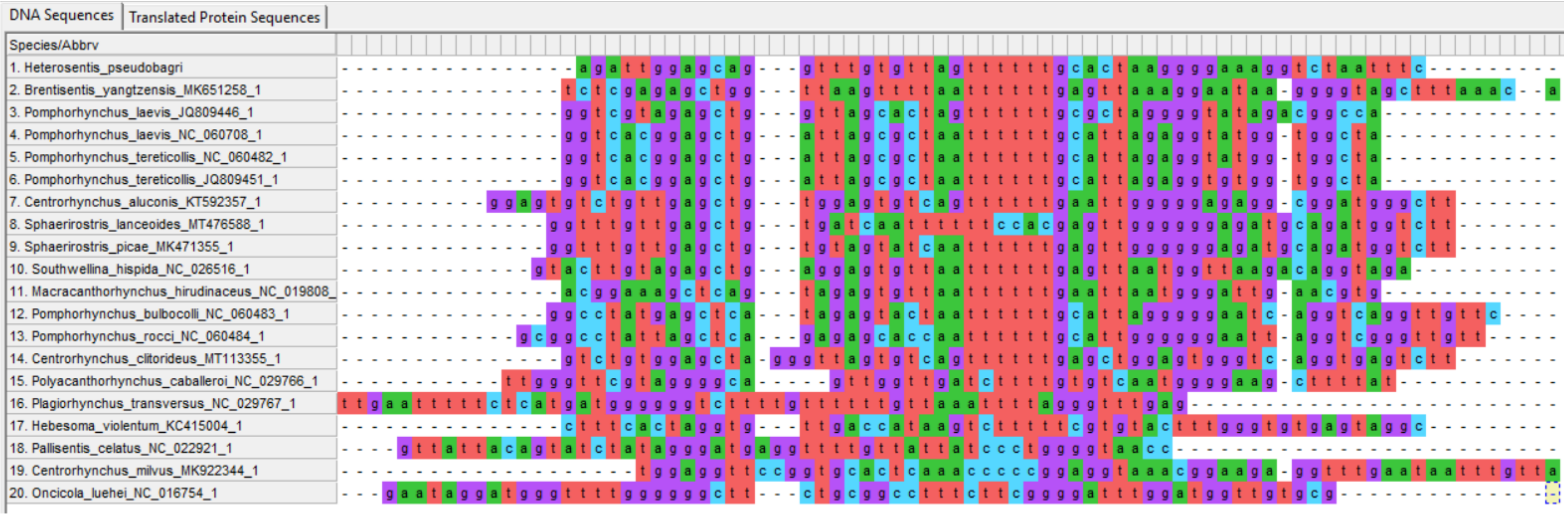

Figure S15. Acanthocephalan *trnK* alignment.

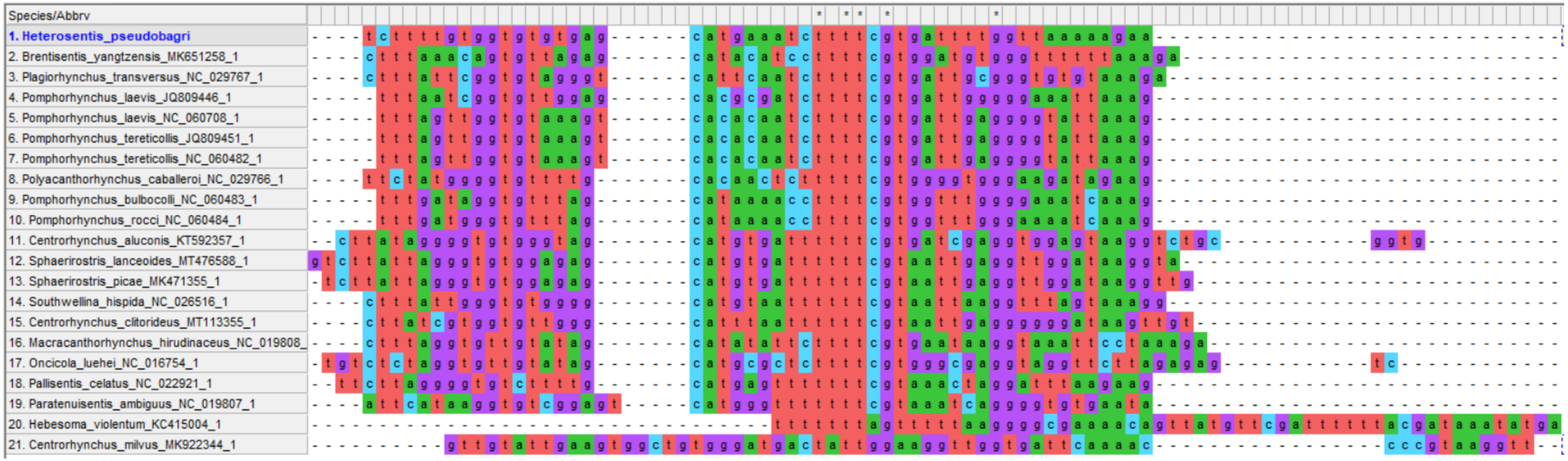

Figure S16. Acanthocephalan *trnE* alignment.

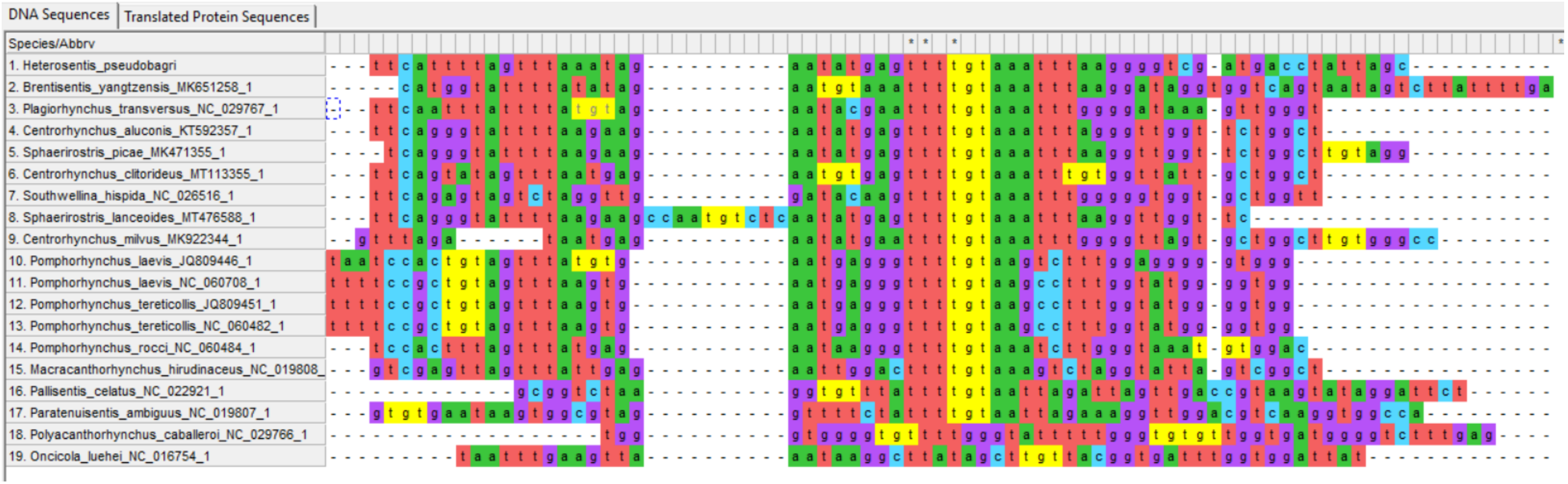

Figure S17. Acanthocephalan *trnT* alignment.

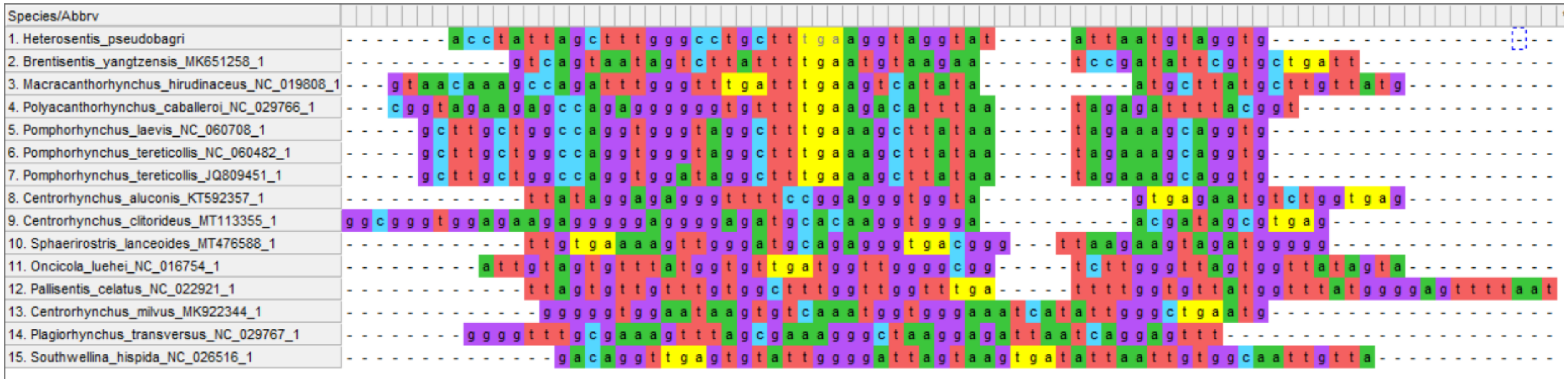

Figure S18. Acanthocephalan *trnS2* alignment.

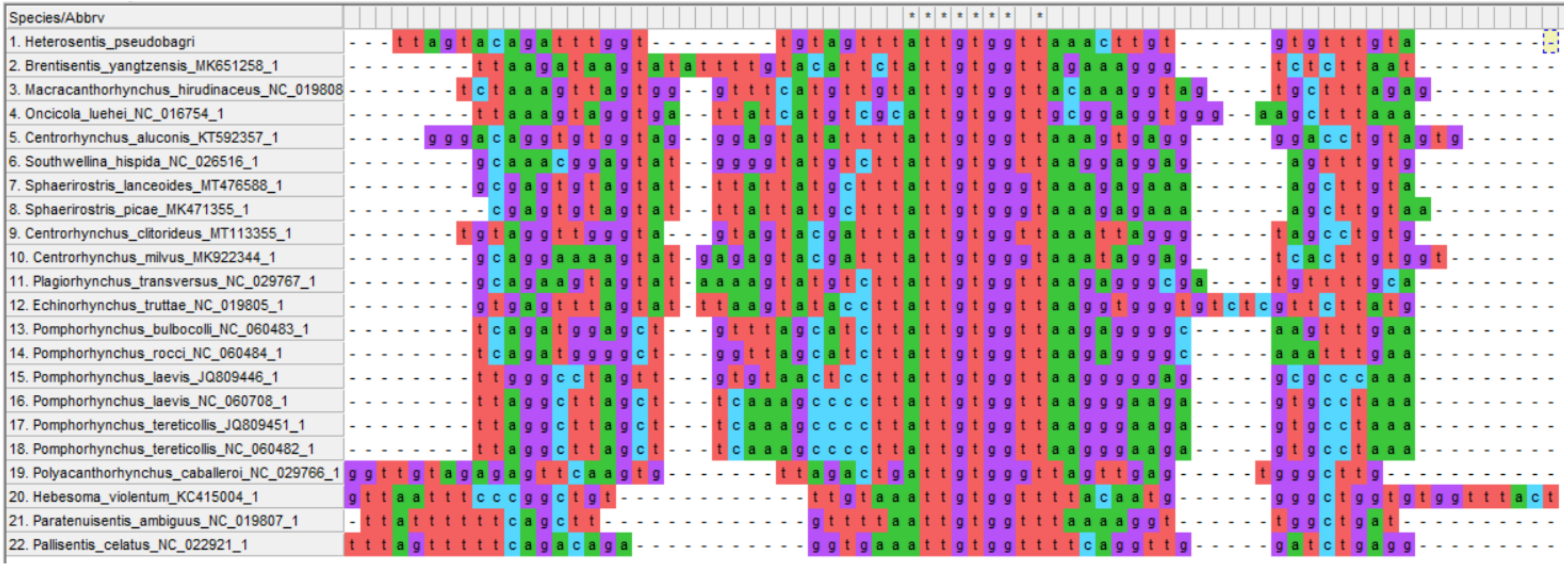

Figure S19. Acanthocephalan *trnH* alignment.

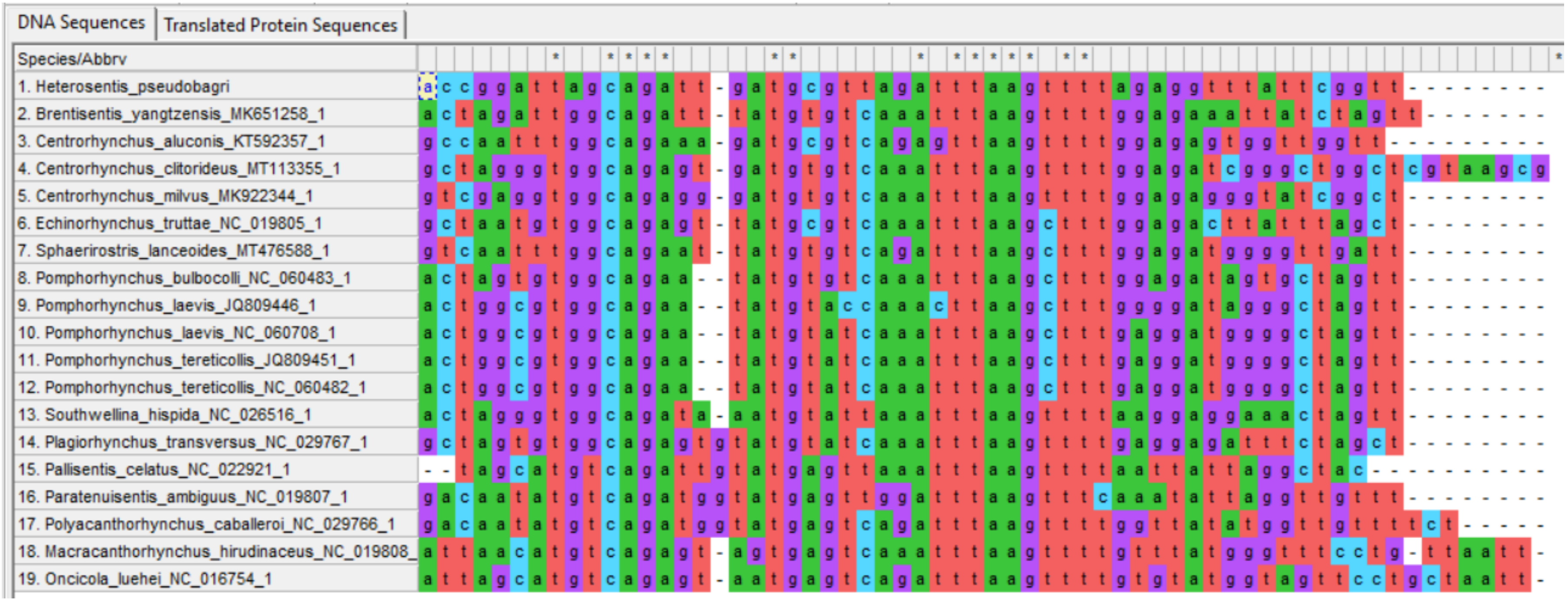

Figure S20. Acanthocephalan *trnL2* alignment.

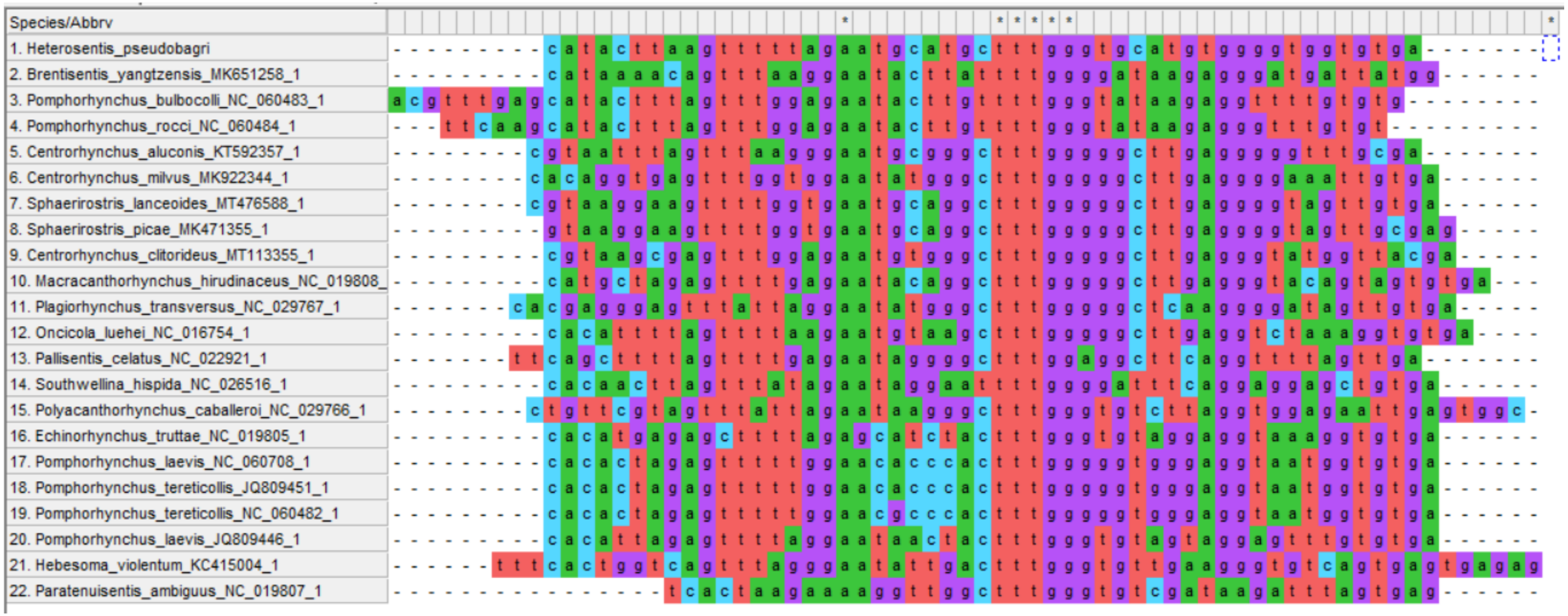

Figure S21. Acanthocephalan *trnP* alignment.

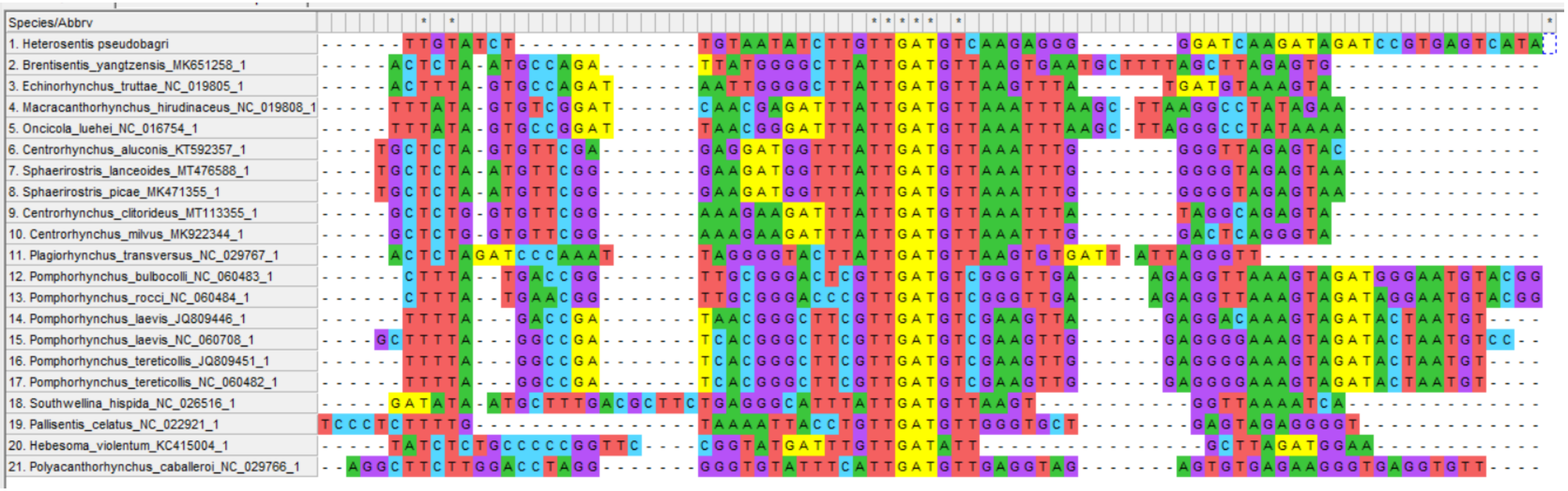

Figure S22. Acanthocephalan *trnI* alignment.

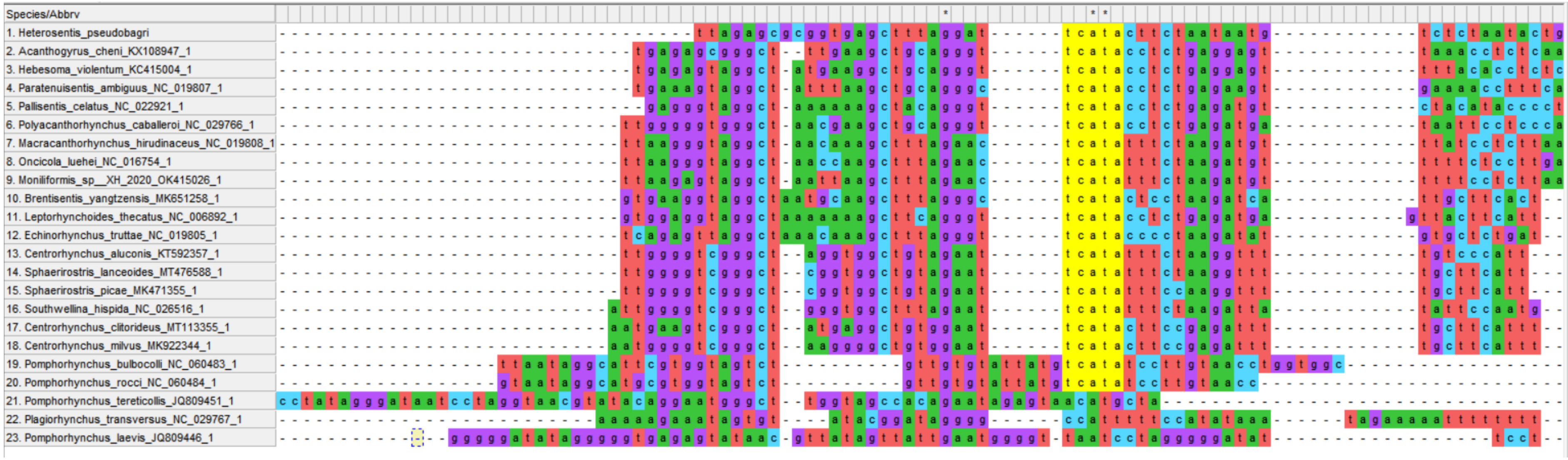

Figure S23. Acanthocephalan trnM alignment.

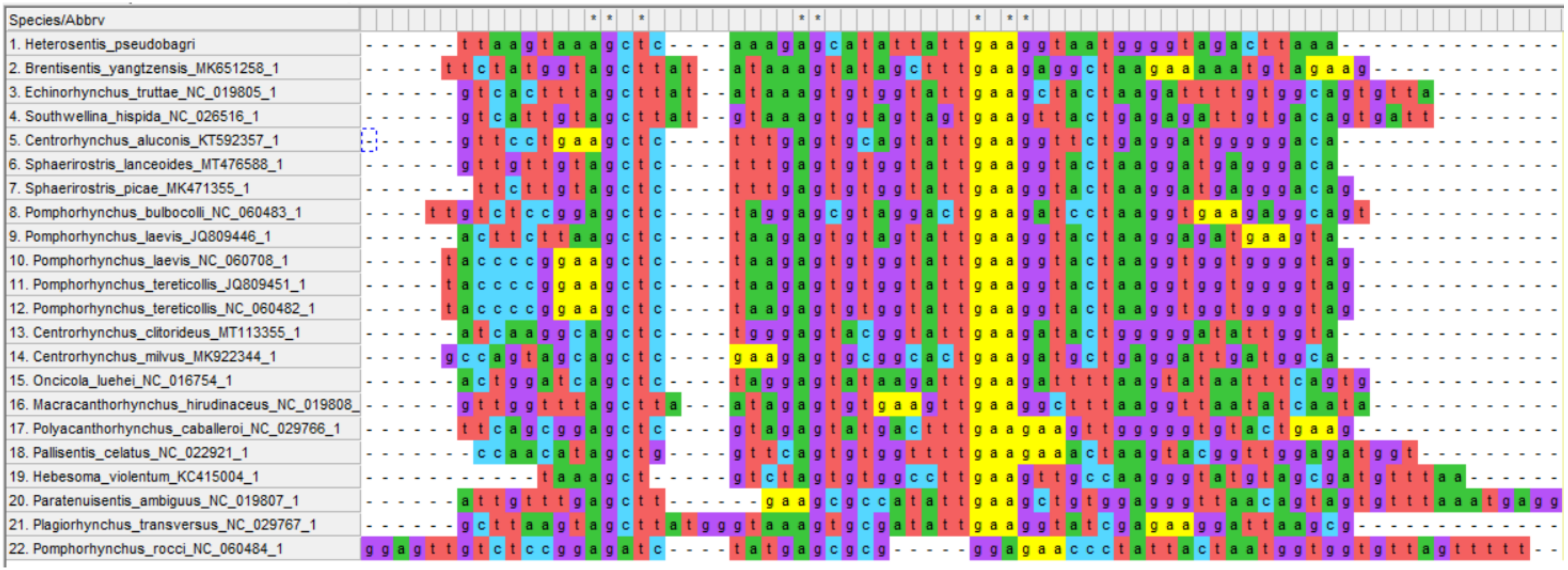

Figure S24. Acanthocephalan trnF alignment.

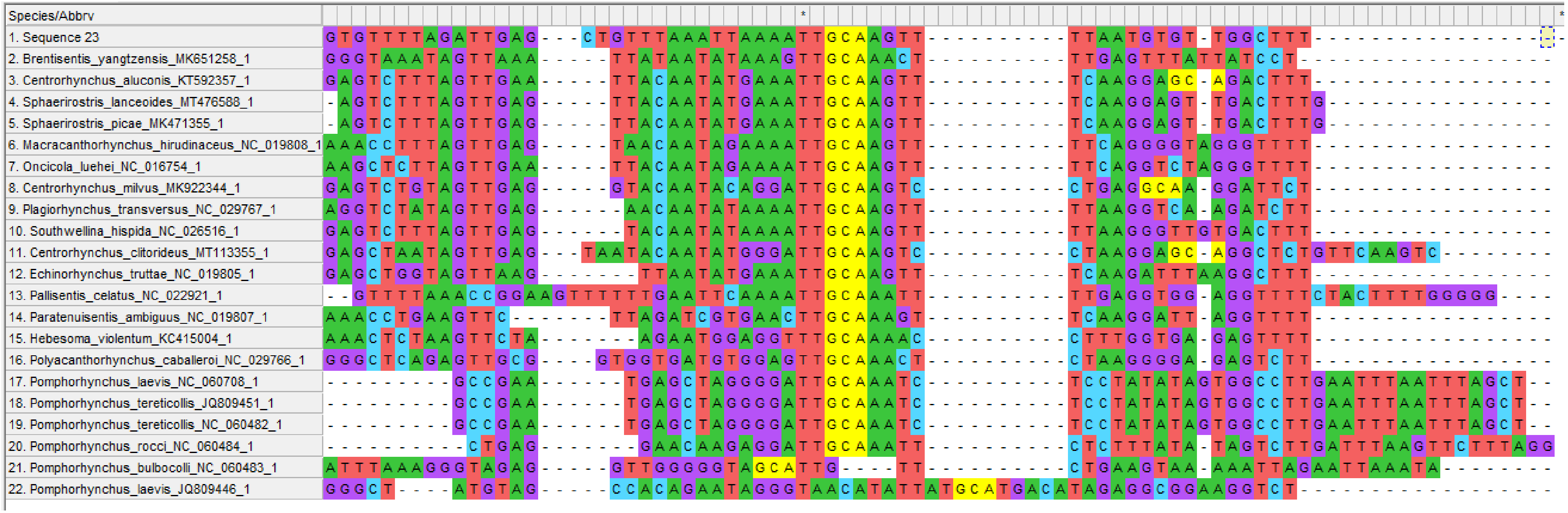

Figure S25. Acanthocephalan trnC alignment.

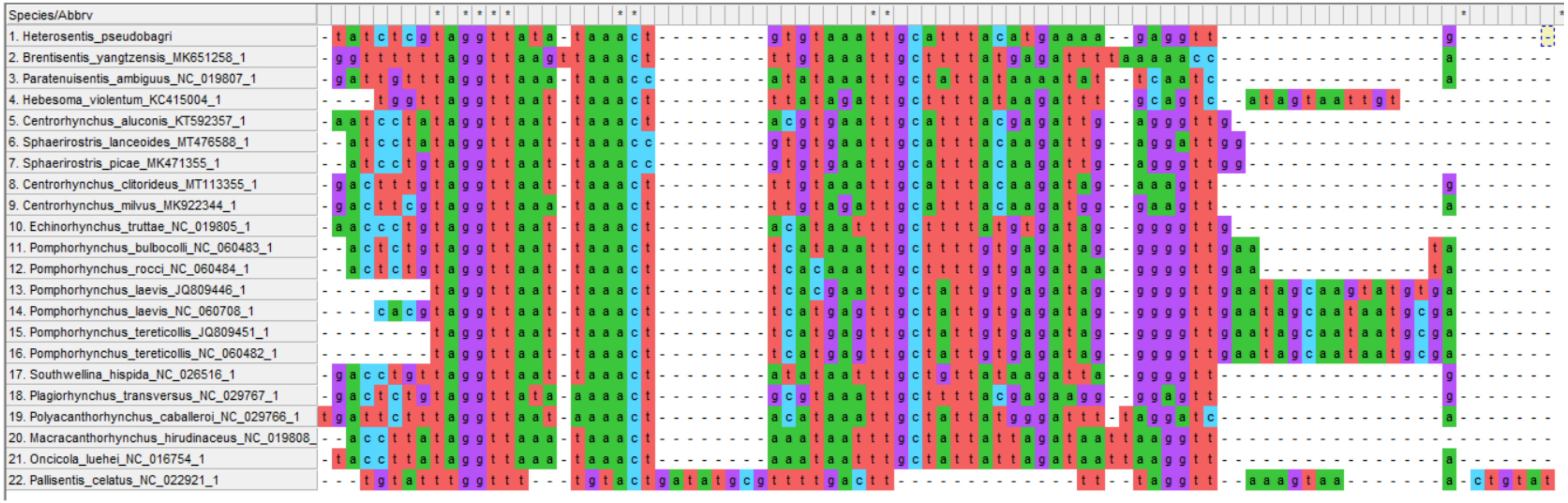

Figure S26. Acanthocephalan trnA alignment.

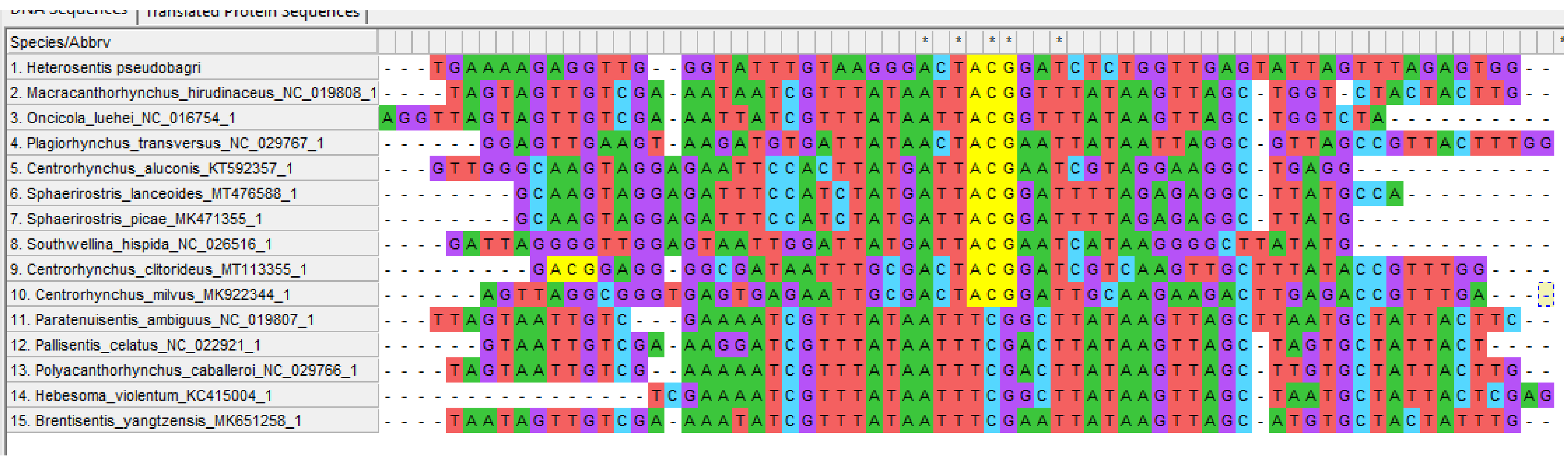

Figure S27. Acanthocephalan trnR alignment.

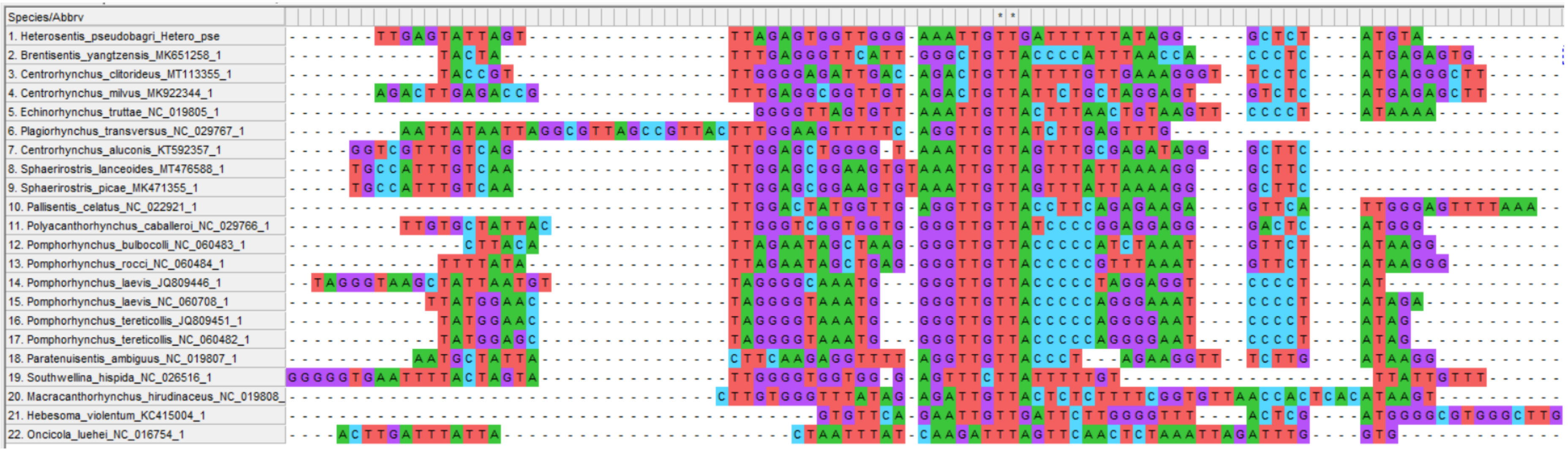

Figure S28. Acanthocephalan trnN alignment.

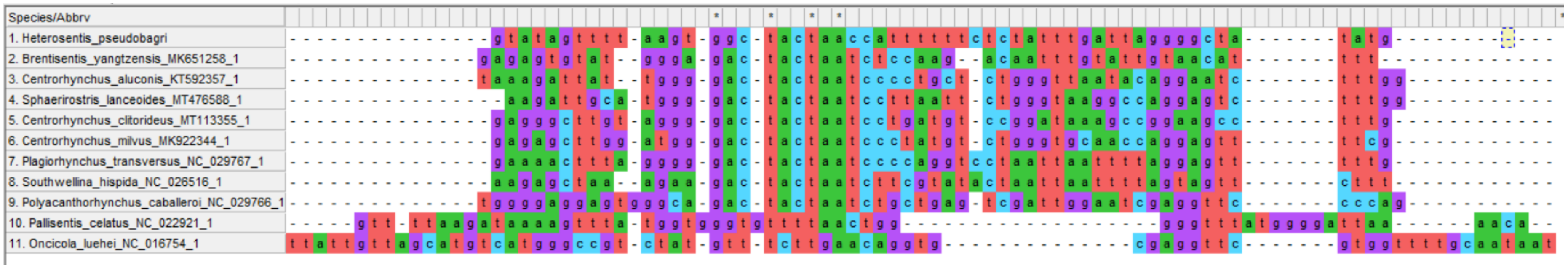

Figure S29. Acanthocephalan trnS1 alignment.

Table S1. Primers used for the amplification and sequencing of the mitogenome of *H. pseudobagri*.

Fragment No.	Gene or region	Primer name	Sequence (5'-3')	Length (bp)
F1	*COX1*	YWF1	GAGCAGAGGTTGTGGCTGCT	327
		YWR1	CTGTAAACATATGATGAGCTC	
F2	*COX1-16S*	YWF2	GGTTGGAGGGAAGTTTGAGG	1677
		YWR2	GTTCTAGGGTCTTTCCGTC	
F3	*16S*	YWF3	GTTGACTATGCTAAGGTAGC	313
		YWR3	TCACGTAACCTACACAGGTC	
F4	*16S-ND4*	YWF4	GGATAACAGGGTAATTAGAG	3392
		YWR4	CAAACCTAAATCATACCTCAC	
F5	*ND4-CYTB*	YWF5	GTTAGGTTTATGGTTAAGGTTCC	3084
		YWR5	AACCCCTGCCACACCTCCTC	
F6	*CYTB*	YWF6	GGAGTTTGGGTGTCAGGAG	516
		YWR6	CCGTAAAATACAGTACACAAAC	
F7	*CYTB-12S*	YWF7	GGAGTTGTTTTTGTTTTTCC	1898
		YWR7	CTATATCTTAATACCTGGGTCTC	
F8	*12S*	YWF8	GTTTAAATTGTGCCAGCGTCAG	400
		YWR8	GTTGACGGGCGATATGTACTC	
F9	*12S-COX2*	YWF9	GATGACATAGAGATTGTATAG	744
		YWR9	CAACCCCACCTGGTTAACC	
F10	*COX2*	YWF10	GCTTTAAGTGTGGATAATCG	200
		YWR10	CCACATAACTCAGAACATTG	
F11	*COX2-COX1*	YWF11	GGTTAAGATTGATTGTATTCCG	2711
		YWR11	GATATACACCTCAGGATGACC	
